# Impact of the introduction of pneumococcal conjugate vaccination on invasive pneumococcal disease and pneumonia in The Gambia: 10 years of population-based surveillance

**DOI:** 10.1016/S1473-3099(20)30880-X

**Published:** 2021-09

**Authors:** The Gambia Pneumococcal Surveillance Group, Grant A Mackenzie, Philip C Hill, David J Jeffries, Malick Ndiaye, Shah M Sahito, Ilias Hossain, Uchendu Uchendu, David Ameh, Oyedeji Adeyemi, Jayani Pathirana, Yekini Olatunji, Baderinwa Abatan, Bilquees S Muhammad, Ebirim Ahameefula, Augustin E Fombah, Banjo Adeshola, Babila G Lobga, Debasish Saha, Roslyn Mackenzie, Aderonke Odutola, Ian D Plumb, Aliu Akano, Bernard E Ebruke, Readon C Ideh, Bankole Kuti, Peter Githua, Emmanuel Olutunde, Ogochukwu Ofordile, Edward Green, Effua Usuf, Henry Badji, Usman NA Ikumapayi, Ahmed Manjang, Rasheed Salaudeen, E David Nsekpong, Sheikh Jarju, Martin Antonio, Sana Sambou, Lamin Ceesay, Yamundow Lowe-Jallow, Sidat Fofana, Momodou Jasseh, Kim Mulholland, Maria Knoll, Orin S Levine, Stephen R Howie, Richard A Adegbola, Brian M Greenwood, Tumani Corrah

**Affiliations:** a Medical Research Council Unit The Gambia at London School of Hygiene & Tropical Medicine, Fajara, The Gambia; b Murdoch Children's Research Institute, Parkville, Melbourne, VIC, Australia; c London School of Hygiene & Tropical Medicine, London, UK; d Department of Paediatrics, University of Melbourne, Melbourne, VIC, Australi; e Centre for International Health, University of Otago, Dunedin, New Zealand; f The National Hospital, Garki, Abuja, Nigeria; g Warwick Medical School, University of Warwick, Coventry, UK; h Ministry of Health, Gambia Government, The Gambia; i Bloomberg School of Public Health, Johns Hopkins University, Baltimore, MD, USA; j Department of Paediatrics: Child and Youth Health, University of Auckland, Auckland, New Zealand; k RAMBICON, Immunisation & Global Health Consulting, Lagos, Nigeria

## Abstract

**Background:**

The Gambia introduced seven-valent pneumococcal conjugate vaccine (PCV7) in August 2009, followed by PCV13 in May, 2011, using a schedule of three primary doses without a booster dose or catch-up immunisation. We aimed to assess the long-term impact of PCV on disease incidence.

**Methods:**

We did 10 years of population-based surveillance for invasive pneumococcal disease (IPD) and WHO defined radiological pneumonia with consolidation in rural Gambia. The surveillance population included all Basse Health and Demographic Surveillance System residents aged 2 months or older. Nurses screened all outpatients and inpatients at all health facilities using standardised criteria for referral. Clinicians then applied criteria for patient investigation. We defined IPD as a compatible illness with isolation of *Streptococcus pneumoniae* from a normally sterile site (cerebrospinal fluid, blood, or pleural fluid). We compared disease incidence between baseline (May 12, 2008–May 11, 2010) and post-vaccine years (2016–2017), in children aged 2 months to 14 years, adjusting for changes in case ascertainment over time.

**Findings:**

We identified 22 728 patients for investigation and detected 342 cases of IPD and 2623 cases of radiological pneumonia. Among children aged 2–59 months, IPD incidence declined from 184 cases per 100 000 person-years to 38 cases per 100 000 person-years, an 80% reduction (95% CI 69–87). Non-pneumococcal bacteraemia incidence did not change significantly over time (incidence rate ratio 0·88; 95% CI, 0·64–1·21). We detected zero cases of vaccine-type IPD in the 2–11 month age group in 2016–17. Incidence of radiological pneumonia decreased by 33% (95% CI 24–40), from 10·5 to 7·0 per 1000 person-years in the 2–59 month age group, while pneumonia hospitalisations declined by 27% (95% CI 22–31). In the 5–14 year age group, IPD incidence declined by 69% (95% CI −28 to 91) and radiological pneumonia by 27% (95% CI −5 to 49).

**Interpretation:**

Routine introduction of PCV13 substantially reduced the incidence of childhood IPD and pneumonia in rural Gambia, including elimination of vaccine-type IPD in infants. Other low-income countries can expect substantial impact from the introduction of PCV13 using a schedule of three primary doses.

**Funding:**

Gavi, The Vaccine Alliance; Bill & Melinda Gates Foundation; UK Medical Research Council; Pfizer Ltd.

## Introduction

Global childhood deaths due to pneumococcal disease are estimated to have fallen from 826 000 in 2000^1^ to 318 000 in 2015.^2^ Introduction of pneumococcal conjugate vaccines (PCV) likely contributed to this decrease. However, despite 60 low-income countries having introduced PCV, data are limited on vaccine impact in these settings.

In South Africa, and Kenya, substantial reductions in invasive pneumococcal disease (IPD) were observed after the introduction of PCV.^3^, ^4^ A schedule with two primary doses and a booster dose was used in South Africa, whereas a three-dose schedule without a booster dose was used in Kenya, accompanied by a catch-up campaign in children younger than 5 years old. However, most low-income countries use a schedule with three primary doses without a booster or catch-up immunisation, and there is little information on this schedule's effectiveness in these countries.

The Gambia introduced PCV7 into its Expanded Program on Immunization (EPI) on August 19, 2009, using three primary doses at ages 2, 3, and 4 months, without catch-up immunisation. PCV13 replaced PCV7 in May, 2011. At the end of 2017, the surveillance system in The Gambia completed a 10-year study to measure the impact of the introduction of PCV, administered according to the standard three-dose schedule adopted widely in low-income countries. We previously reported the impact of routine infant vaccination with PCV 4 years after its introduction and documented modest reductions in IPD and radiological pneumonia.^5^, ^6^ We report now on vaccine impact 8 years after the introduction of PCV.

**Research in context**

**Evidence before this study**

In high-income and middle-income countries the introduction of pneumococcal conjugate vaccine (PCV) into routine immunisation programmes has led to significant reductions in pneumonia hospitalisations and invasive pneumococcal disease (IPD) because of vaccine serotypes. We did a systematic literature search of studies of PCV10 and PCV13 effectiveness or impact in low-income countries on PubMed, Embase, and Web of Science for the period Jan 1, 2008, to Aug 31, 2020. We searched using the terms “pneumococcal vaccines” OR “vaccines, pneumococcal conjugate” AND “meningitis”, “sepsis”, “septic(a)emia”, “bacter(a)emia”, “invasive pneumococcal disease”, “pneumonia” AND “impact” OR “effectiveness”. We searched for prospective population-based studies that included at least 1 year of data before PCV introduction and at least 2 years of data after vaccine introduction. Two publications reported the impact on IPD and pneumonia in The Gambia, 4 and 5 years, after the introduction of PCV with the standard schedule of three primary doses without a booster dose or catch-up immunisation. In the 2–59 month age group, all IPD declined by 55% and radiological pneumonia declined by 23%. Two Kenyan publications reported the impact of PCV10 introduced using the standard three-dose schedule with catch-up immunisation to 5 years of age. In children younger than 5 years, all IPD declined by 68% and radiological pneumonia declined by 48%. In Burkina Faso, modest reductions in pneumococcal meningitis were noted 3–4 years after the introduction of PCV13, although preexisting temporal trends precluded precise estimation of vaccine impact. Despite pneumococcal disease being a major cause of child mortality, and the introduction of PCV in 60 low-income countries, there are few prospective population-based data reporting PCV impact in such settings. In particular, there are no data reporting long-term impact in a low-income country where PCV was introduced with the standard schedule of three primary doses without a booster dose and without catch-up immunisation.

**Added value of this study**

This study provides the first evidence of long-term PCV impact when immunisation is introduced with the standard three-dose schedule without a booster or catch-up dose, as is the norm in almost all low-income countries. 8 years after the introduction of PCV7 or PCV13, this 10-year population based surveillance study observed a 92% reduction in the incidence of IPD due to vaccine-types in the 2–59 month age group, an 80% reduction in all IPD (greater than the 55% reduction observed after 4 years), and no significant change in non-vaccine type IPD. In the final 2 years of the study, we observed zero cases of vaccine-type IPD in infants. The incidence of radiological pneumonia declined by 33% (greater than the 24% reduction after 5 years) and severe hypoxic pneumonia declined by 60%. The 27% reduction in hospitalised clinical pneumonia was greater than the 8% reduction previously reported. There was no change in the incidence of the control condition of non-pneumococcal bacteraemia, suggesting the reductions in the disease endpoints were related to the introduction of PCV. The full impact of vaccination with PCV took 8 years to develop.

**Implications of all the available evidence**

Our findings are widely relevant as the data indicate that the routine introduction and long-term use of PCV13 in low-income countries will substantially reduce rates of invasive pneumococcal disease and severe pneumonia, where the burden is greatest. Countries should consider the use of catch-up immunisation when PCV is introduced and implement long-term surveillance to measure the full impact of the introduction of PCV. These results should encourage low-income countries that have not yet introduced PCV to do so, and those that are using PCV should maintain budget support for their programmes as they transition from financial support from Gavi, the Vaccine Alliance.

## Methods

### Surveillance and population

We did population-based surveillance for suspected pneumonia, septicaemia, and meningitis in the Basse Health and Demographic Surveillance System^7^ (BHDSS, appendix p 6) between May 12, 2008, and Dec 31, 2017. The surveillance population included all BHDSS residents aged 2 months or older. Here we report findings restricted to children aged 2 months to 14 years. The total population of the BHDSS in 2017 was 181 740, including 85 279 aged 2 months to 14 years.

### Endpoints

The primary endpoints were IPD and radiological pneumonia with consolidation, as defined by WHO.^8^ We defined IPD as a compatible illness with isolation of *Streptococcus pneumoniae* from a normally sterile site (cerebrospinal fluid, blood, or pleural fluid). Secondary endpoints of clinical and hypoxic pneumonia were defined as in our previous report.^6^ IPD outcomes were further categorised according to PCV13 serotype (1, 3, 4, 5, 6A, 6B, 7F, 9V, 14, 18C, 19A, 19F, and 23F) or non-PCV13 serotype. We do not present impact estimates on pneumococcal pneumonia because it is a subgroup of IPD. We defined bronchiolitis and vaccine failure as previously reported (see full definitions in the appendix).^5^, ^6^

### Procedures

The surveillance methods have been described previously.^5^, ^6^, ^7^ In brief, nurses assessed all outpatients and inpatients at all health facilities in the BHDSS using standardised criteria for referral to clinicians in Basse (appendix p 24). For referred patients, clinicians then applied standardised criteria to identify patients with suspected pneumonia, septicaemia, or meningitis and requested blood culture, lumbar puncture, or chest radiography (appendix pp 25–26). Aspiration of pleural fluid or lung aspiration was done for selected patients. Each year, rapid malaria tests (ICT Diagnostics, Cape Town, South Africa) were done on all enrolled patients from August to December (the malaria transmission season). Surveillance was interrupted between Oct 5 and Nov 3, 2010 because of flooding. Radiographs were done according to WHO recommendations^8^ and interpreted by two independent reviewers, with readings discordant for radiological endpoint consolidation resolved by a third reviewer.

Specimens were processed in Basse with standard methods.^9^ *S pneumoniae* was identified by colony morphology and optochin sensitivity. Pneumococcal isolates were serotyped at the WHO Regional Reference Laboratory (Medical Research Council Unit The Gambia [MRC], Fajara, The Gambia), using latex agglutination with factor-specific and group-specific antisera.^5^ Serotypes 6A and 6B were differentiated from 6C by PCR.^10^ Basse and Fajara laboratories submit to external quality assurance (UK National External Quality Assessment Service [Sheffield, UK] and OneWorld Accuracy International, Canada).

The Gambia Government/MRC Joint Ethics Committee (number 1087) and the London School of Hygiene & Tropical Medicine ethics committee approved the study. Parents or guardians gave written informed consent for all surveillance procedures.

### Statistical analysis

We calculated annual incidence by dividing the number of cases by midpoint population estimates obtained each year from the BHDSS. To calculate incidence in 2008 and 2010, we extrapolated cases and assigned serotype groupings for the unobserved periods as described previously.^5^, ^6^

As in our previous IPD analysis,^5^ we adjusted for changes over time in age-specific numbers of patients eligible for investigation per unit population by adjusting annual IPD counts, assuming the same serotype distribution as the observed cases each year. We adjusted annual, age-specific counts of IPD to the mean rate of enrolment of patients eligible for investigation during the study period. As in our previous pneumonia analysis,^6^ we adjusted annual case counts for age-specific changes over time in the number of patients referred to clinicians per unit population. We adjusted annual, age-specific counts of pneumonia to the mean rate of referral during the study period. Thus, interpretation of adjusted analyses assumes a constant rate of enrolment of patients eligible for investigation and a constant rate of referral to clinicians during the observation period.

Consistent with our previous analyses,^5^, ^6^ we calculated the ratio of the incidence in the last 2 years of surveillance (2016–17) compared to the baseline first 2 years (May 12, 2008, to May 11, 2010). We used the Poisson distribution to calculate incidence rate ratios (IRR) with 95% CI. CIs were inflated allowing for over-dispersion in the 2–23 month (IPD), 2–4 year (radiological pneumonia), and 5–14 year (IPD) age groups, estimated from an individual-level Poisson regression analysis of 2008–09 data.

To investigate potential bias due to temporal changes in health care seeking, patient investigation, or confounding due to secular trends in epidemic serotypes we did three a priori stratified analyses, excluding outpatients, cases identified by lung aspiration alone, and cases caused by serotype 1 or 5. To assess the effect of temporal secular trends related to bacterial disease, as control endpoints, we evaluated changes in the incidence of non-pneumococcal bacteraemia and non-pneumococcal pneumonia. We also evaluated temporal changes in the prevalence of contaminated blood cultures, malnutrition, and malaria over time.^5^, ^6^ Statistical significance was set at a p value less than 0·05. The data were analysed with Stata (version 15.1) and MATLAB (version R2015a).

### Role of the funding source

The funders had no role in the study design, data collection, data analysis, data interpretation, or writing of the report.

## Results

Coverage of one dose of PCV7 in the 12–23 month age group at the end of 2009 was 10%. Coverage of at least two doses of PCV13 reached plateaus in 2013 of around 55% and 75% in the 2–11 and 12–23 month age groups, respectively (appendix p 7). In the 2–4 year age group, coverage of at least two doses in 2016–17 was about 78%, greater than the 50% coverage in the final 2 years of surveillance in our earlier report;^6^ coverage in the 5–14 year age group was about 10%. During the period 2012–17, coverage of two doses before the ages of 4, 5, and 6 months was 42%, 67%, and 77%, respectively, and coverage of three doses before 5, 6, and 7 months of age was 29%, 50%, and 63%, respectively. In 2016–17 coverage of three doses before 6 months of age was 59%. We assumed that second or third doses of PCV that were received more than 24 weeks after the previous dose might act as a booster rather than primary dose. During 2012–17, 2% of infants received their second dose more than 24 weeks after their first dose and 3% received their third dose more than 24 weeks after their second dose. The low proportion of infants who received substantially delayed second or third doses of PCV suggest that our results relate to programmatic use of a three-dose schedule without booster or catch-up dose.

**Figure 1:**
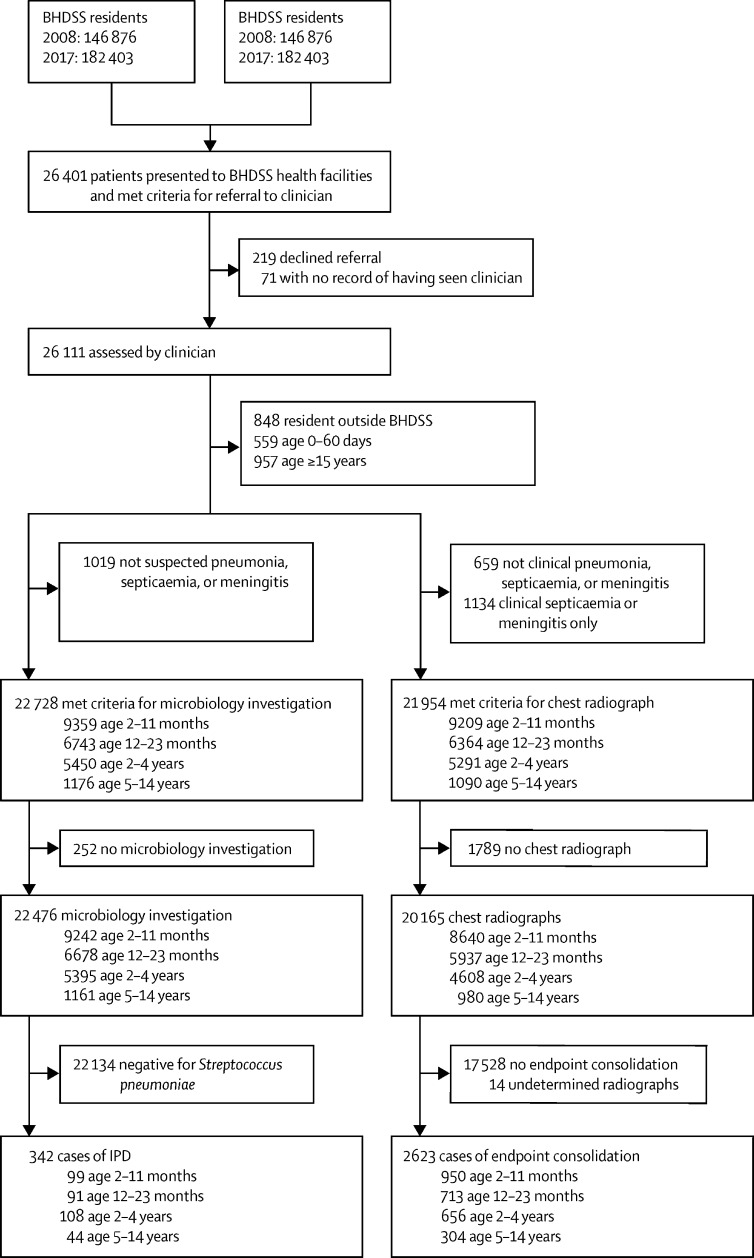
Study profile BHDSS=Basse Health and Demographic Surveillance System

A total of 26 401 patients were screened; 22 728 patients aged 2 months to 14 years met criteria for microbiological investigation and 21 954 met criteria for radiological investigation (figure 1). Surveillance was done at a consistently high level (appendix pp 8–12). We identified 342 cases of IPD and 2623 cases of radiological pneumonia. 29 IPD cases (8·5%) died, with 14% mortality (14 of 99) in the first year of life (Table 1, Table 2). By far the largest number of pneumonia deaths occurred in those with clinical pneumonia, but mortality in children with clinical (2·1%) or radiological pneumonia (2·4%) was much lower than mortality in cases of pneumococcal pneumonia (8·6%) or hypoxic pneumonia (12·1%).

**Table 1:** Characteristics of cases of IPD by serotype between May 12, 2008, and Dec 31, 2017

		**All IPD (342)**	PCV7 (73)*	PCV13 only (139)*	Non-vaccine type (128)*
Age					
	2–11 months	99 (29%)	17 (23%)	12 (9%)	67 (52%)
	12–23 months	91 (27%)	27 (37%)	36 (26%)	29 (23%)
	2–4 years	108 (32%)	26 (36%)	55 (40%)	27 (21%)
	5–14 years	44 (13%)	3 (4%)	36 (26%)	5 (4%)
Male		196 (57%)	41 (56%)	82 (59%)	70 (55%)
Female		146 (43%)	32 (44%)	57 (41%)	58 (45%)
Diagnostic category†					
	Meningitis	40 (12%)	11 (15%)	10 (7%)	19 (15%)
	Septicaemia	101 (30%)	11 (15%)	34 (24%)	54 (42%)
	Pneumonia	201 (59%)	51 (70%)	95 (68%)	55 (43%)
Pathology					
	Meningitis	24 (7%)	8 (11%)	3 (2%)	13 (10%)
	Non-meningitis	318 (93%)	65 (89%)	136 (98%)	115 (90%)
Weight-for-height z-score‡ less than −3		52/298 (17%)	14/70 (20%)	11/103 (11%)	26/123 (21%)
Treated as inpatient		304 (89%)	63 (86%)	132 (95%)	21 (84%)
Deaths		29 (8%)	6 (8%)	8 (6%)	15 (12%)
	Proven meningitis	8 (2%)	4 (5%)	0	4 (3%)
	Non-meningitis	21 (6%)	2 (3%)	8 (6%)	11 (9%)

Data are n (%). IPD=invasive pneumococcal disease. PCV7=serotypes covered by the 7-valent PCV: 4, 6B, 9V, 14, 18C, 19F, 23F, and cross-reactive 6A. PCV13 only=serotypes covered only by the 13-valent PCV: 1, 3, 5, 7F, and 19A. Non-vaccine type=serotypes not covered by PCV13.

* The number of episodes PCV7, PCV13 only, and non-vaccine type IPD do not sum to 342 cases; in three cases there were two different serotypes detected in different samples and in five cases the isolate could not be serotyped.

† Categories of surveillance diagnosis (appendix) are mutually exclusive and ranked by severity; meningitis most severe, then septicaemia, and pneumonia.

‡ Defined using the 2006 WHO anthropometry standards, age 2–59 months.

**Table 2:** Characteristics of cases of pneumonia by type between May 12, 2008, and Dec 31, 2017

		Clinical pneumonia*(21 954)	**Radiological pneumonia**†**(2623)**	**Pneumococcal pneumonia**‡**(337)**	**Hypoxic clinical pneumonia**§**(801)**
Age					
	2–11 months	9209 (42·0%)	950 (36·2%)	97 (28·8%)	419 (52·3%)
	12–23 months	6364 (28·0%)	713 (27·2%)	89 (26·4%)	210 (26·2%)
	2–4 years	5291 (24·1%)	656 (25·0%)	107 (31·8%)	145 (18·1%)
	5–14 years	1090 (5·0%)	304 (11·6%)	44 (13·1%)	27 (3·4%)
Male		12 334 (56·2%)	1427 (54·4%)	193 (57·3%)	415 (51·8%)
Female		9620 (43·8%)	1196 (45·6%)	144 (42·7%)	386 (48·2%)
Weight-for-height z-score¶ less than −3		2447/20 864 (11·7%)	337/2319 (14·5%)	50/293 (17·1%)	129/774 (16·7%)
Treated as inpatient		12 812 (58·4%)	2166 (82·6%)	301 (89·3%)	749 (93·5%)
Mortality		467 (2·1%)	63 (2·4%)	29 (8·6%)	97 (12·1%)

Data are n (%) or n/N (%).

* Defined as acute cough or shortness of breath with raised respiratory rate for age, inability to feed or sit, reduced conscious state, convulsions, lower chest wall in-drawing, peripheral arterial oxygen saturation <93%, dull chest percussion note, or bronchial breathing on auscultation.

† Defined as the WHO standard for childhood radiological pneumonia with consolidation.

‡ Defined as clinical pneumonia with isolation of *Streptococcus pneumoniae* from a normally sterile site.

§ Defined as clinical pneumonia with peripheral arterial oxygen saturation <90% at presentation.

¶ Defined by the 2006 WHO anthropometry standards, age 2–59 months.

**Figure 2:**
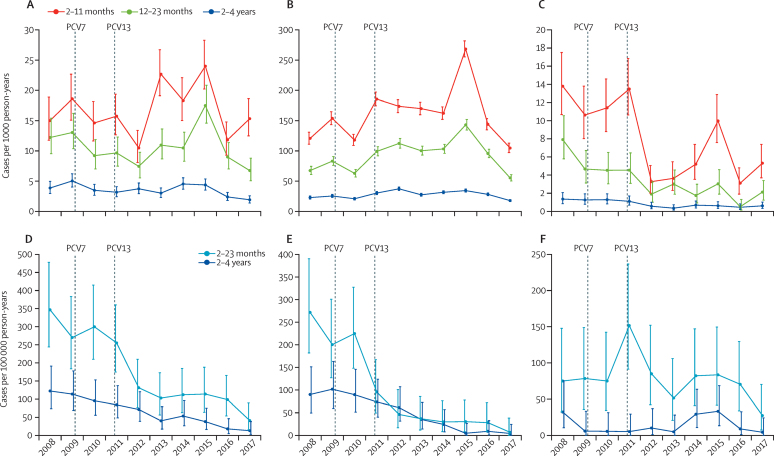
Adjusted annual incidence of invasive pneumococcal disease and pneumonia endpoints in Basse Health and Demographic Surveillance System 2008–17 Radiological pneumonia with consolidation (A). Clinical pneumonia (B). Hypoxic pneumonia (C). Invasive pneumococcal disease (D). Vaccine-type invasive pneumococcal disease (E). Non-vaccine type invasive pneumococcal disease (F).

Crude and adjusted impact estimates for the primary endpoints in the 2–59 month age group differed by 10% or less, indicating no significant confounding due to changes in case ascertainment over time (Table 3, Table 4).^11^ Comparing the baseline and 2016–17 periods, the adjusted incidence of IPD in the 2–59 month age group declined from 184 cases per 100 000 person-years to 38 cases per 100 000 person-years, an 80% reduction (95% CI 69–87; table 3, figure 2). The adjusted incidence of PCV13-type IPD declined by 92% (95% CI 83–96), from 147 cases per 100 000 person-years to 12 cases per 100 000 person-years. The adjusted incidence of IPD caused by non-PCV13 serotypes was 37 cases per 100 000 person-years at baseline compared with 24 cases per 100 000 person years in 2016–17, a 31% reduction that was not significant (95% CI −29 to 64).

**Table 3:** Number of cases and incidence of IPD in the baseline period and 2016–17 after vaccine introduction

	**Baseline adjusted (crude) cases**	**Baseline adjusted (crude) incidence per 100 000 person-years**	**2016–17 adjusted (crude) cases**	**2016–17 adjusted (crude) incidence per 100 000 person-years**	**Crude IRR 2016–17 *vs* baseline (95% CI)**	**Adjusted IRR 2016–17 *vs* baseline (95% CI)**
**Age 2–23 months**						
All	62 (50)	281 (226)	20 (18)	71 (63)	0·28 (0·16–0·48)	0·25 (0·15–0·42)
PCV7	29 (23)	129 (104)	2 (2)	7 (7)	0·07 (0·02–0·30)	0·06 (0·01–0·23)
PCV13	47 (38)	213 (171)	6 (5)	20 (17)	0·10 (0·04–0·26)	0·09 (0·04–0·24)
PCV13 only	20 (16)	89 (72)	4 (3)	12 (10)	0·14 (0·04–0·51)	0·14 (0·05–0·46)
Non-vaccine type	15 (12)	67 (54)	15 (13)	51 (45)	0·83 (0·37–1·87)	0·76 (0·37–1·61)
**Age 2–4 years**						
All	38 (33)	118 (102)	7 (6)	16 (13)	0·13 (0·04–0·31)	0·13 (0·06–0·30)
PCV7	15 (13)	46 (40)	1 (1)	2 (2)	0·05 (0·001–0·37)	0·05 (0·007–0·37)
PCV13	33 (29)	103 (90)	4 (3)	8 (7)	0·07 (0·01–0·24)	0·08 (0·03–0·23)
PCV13 only	19 (17)	60 (53)	3 (2)	6 (4)	0·08 (0·009–0·35)	0·09 (0·02–0·34)
Non-vaccine type	5 (4)	15 (12)	4 (3)	8 (6)	0·54 (0·08–3·16)	0·54 (0·14–2·11)
**Age 2–59 months**						
All	100 (83)	184 (153)	28 (24)	38 (32)	0·21 (0·14–0·33)	0·20 (0·13–0·31)
PCV7	43 (36)	79 (66)	3 (3)	4 (4)	0·06 (0·02–0·20)	0·05 (0·02–0·17)
PCV13	80 (67)	147 (123)	9 (8)	12 (11)	0·09 (0·04–0·18)	0·08 (0·04–0·17)
PCV13 only	39 (33)	72 (61)	6 (5)	8 (7)	0·11 (0·04–0·29)	0·11 (0·05–0·27)
Non-vaccine type	20 (16)	37 (29)	18 (16)	24 (22)	0·74 (0·37–1·47)	0·69 (0·36–1·29)
**Age 5–14 years**						
All	9 (10)	10 (12)	4 (3)	3 (3)	0·23 (0·05–0·98)	0·31 (0·09–1·28)
PCV7	0 (0)	..	0 (0)	..	..	..
PCV13	8 (9)	9 (11)	1 (1)	1 (1)	0·09 (0·008–0·87)	0·11 (0·009–1·00)
PCV13 only	8 (9)	9 (11)	1 (1)	1 (1)	0·09 (0·008–0·87)	0·11 (0·009–1·00)
Non-vaccine type	1 (1)	1 (1)	2 (2)	2 (2)	1·53 (0·10–22·9)	1·53 (0·10–22·9)

The baseline period was May 12, 2008 to May 11, 2010. Numbers of serotype-specific IPD cases may not sum to the number of all IPD cases due to five isolates that could not be serotyped. Case counts are adjusted for trends in patients eligible for investigation and rounded to the nearest integer. CIs calculated taking into account over-dispersed Poisson distributions in the 2–23 months and 5–14 years age groups. IPD=invasive pneumococcal disease. IRR=incidence rate ratio. PCV7=serotypes covered by PCV7: 4, 6B, 9V, 14, 18C, 19F, 23F, and cross-reactive 6A. PCV13=serotypes covered by PCV13. PCV13 only=serotypes covered by PCV13 but not PCV7: 1, 3, 5, 7F, and 19A. Non-vaccine type=serotypes not covered by PCV13.

**Table 4:** Numbers of cases and incidence of pneumonia endpoints in the baseline period and in 2016–17 after vaccine introduction

	**Baseline adjusted (crude) cases**	**Baseline adjusted (crude) incidence per 1000 person-years**	**2016–17 adjusted (crude) cases**	**2016–17 adjusted (crude) incidence per 1000 person-years**	**Crude IRR 2016–17 *vs* baseline (95% CI)**	**Adjusted IRR 2016–17 *vs* baseline (95% CI)**
**Age 2–11 months**						
Radiological pneumonia with consolidation	215 (180)	20·7 (17·3)	233 (177)	17·9 (13·6)	0·79 (0·64–0·97)	0·87 (0·72–1·04)
Hospitalised radiological pneumonia with consolidation	184 (154)	17·7 (14·8)	178 (137)	13·7 (10·5)	0·71 (0·56–0·90)	0·77 (0·63–0·95)
Clinical pneumonia	1708 (1426)	164·2 (137·1)	2021 (1615)	155·3 (124·1)	0·91 (0·84–0·97)	0·95 (0·89–1·01)
Hospitalised clinical pneumonia	1095 (915)	105·3 (88·0)	1030 (823)	79·1 (63·2)	0·72 (0·65–0·79)	0·75 (0·69–0·82)
Hypoxic clinical pneumonia	134 (112)	12·9 (10·8)	74 (55)	5·7 (4·2)	0·39 (0·28–0·54)	0·44 (0·33–0·59)
**Age 12–23 months**						
Radiological pneumonia with consolidation	189 (149)	15·3 (12·1)	161 (124)	10·2 (7·9)	0·65 (0·51–0·83)	0·67 (0·54–0·82)
Hospitalised radiological pneumonia with consolidation	156 (123)	12·6 (9·9)	128 (100)	8·1 (6·3)	0·64 (0·49–0·83)	0·64 (0·51–0·82)
Clinical pneumonia	1149 (906)	92·9 (73·3)	1490 (1187)	94·4 (75·2)	1·03 (0·94–1·12)	1·02 (0·94–1·10)
Hospitalised clinical pneumonia	687 (542)	55·6 (43·8)	691 (533)	43·8 (33·8)	0·77 (0·68–0·87)	0·79 (0·71–0·88)
Hypoxic clinical pneumonia	82 (65)	6·6 (5·3)	32 (21)	2·0 (1·3)	0·25 (0·15–0·41)	0·31 (0·20–0·46)
**Age 2–4 years**						
Radiological pneumonia with consolidation	172 (148)	5·3 (4·6)	125 (97)	2·8 (2·1)	0·47 (0·35–0·62)	0·52 (0·40–0·67)
Hospitalised radiological pneumonia with consolidation	138 (119)	4·3 (3·7)	105 (81)	2·3 (1·8)	0·49 (0·36–0·66)	0·54 (0·41–0·72)
Clinical pneumonia	930 (796)	28·8 (24·7)	1307 (1034)	28·9 (22·9)	0·93 (0·84–1·03)	1·00 (0·91–1·10)
Hospitalised clinical pneumonia	583 (499)	18·1 (15·5)	609 (475)	13·5 (10·5)	0·68 (0·59–0·78)	0·75 (0·66–0·84)
Hypoxic clinical pneumonia	44 (38)	1·4 (1·2)	32 (24)	0·7 (0·5)	0·45 (0·26–0·79)	0·52 (0·31–0·86)
**Age 2–59 months**						
Radiological pneumonia with consolidation	575 (477)	10·5 (8·7)	519 (398)	7·0 (5·4)	0·62 (0·54–0·71)	0·67 (0·60–0·76)
Hospitalised radiological pneumonia with consolidation	478 (396)	8·7 (7·2)	411 (318)	5·6 (4·3)	0·60 (0·51–0·69)	0·64 (0·56–0·73)
Clinical pneumonia	3782 (3124)	68·8 (56·8)	4815 (3834)	65·1 (51·8)	0·91 (0·87–0·96)	0·95 (0·91–0·99)
Hospitalised clinical pneumonia	2368 (1958)	43·1 (35·6)	2332 (1832)	31·5 (24·8)	0·70 (0·65–0·74)	0·73 (0·69–0·78)
Hypoxic clinical pneumonia	261 (215)	4·7 (3·9)	139 (100)	1·9 (1·4)	0·35 (0·27–0·44)	0·40 (0·32–0·49)
**Age 5–14 years**						
Radiological pneumonia with consolidation	59 (66)	0·7 (0·8)	56 (41)	0·5 (0·4)	0·48 (0·32–0·70)	0·73 (0·51–1·05)
Hospitalised radiological pneumonia with consolidation	48 (54)	0·6 (0·6)	46 (34)	0·4 (0·3)	0·48 (0·31–0·74)	0·73 (0·49–1·10)
Clinical pneumonia	203 (233)	2·4 (2·7)	264 (189)	2·4 (1·7)	0·62 (0·51–0·75)	1·00 (0·86–1·20)
Hospitalised clinical pneumonia	110 (127)	1·3 (1·5)	81 (59)	0·7 (0·5)	0·36 (0·26–0·49)	0·57 (0·42–0·75)
Hypoxic clinical pneumonia	7 (8)	0·08 (0·09)	2 (2)	0·02 (0·02)	0·19 (0·04–0·90)	0·27 (0·06–1·19)

The baseline period was May 12, 2008, to May 11, 2010. Case counts are adjusted for temporal trends in the rate of patients meeting criteria for referral to surveillance clinicians and rounded to the nearest integer. CIs are calculated using an inflated variance due to over-dispersion in the 2–4 years age group. IRR=incidence rate ratio.

In the 5–14 year age group, crude and adjusted impact estimates differed by more than 10%, suggesting significant confounding due to temporal changes in case ascertainment.^11^ The adjusted incidence of all IPD among those aged 5–14 years declined by 69% (95% CI −28 to 91), from 10 cases per 100 000 person-years to 3 cases per 100 000 person-years (table 3, appendix p 13). In contrast to our earlier report,^5^ the adjusted incidence of PCV13-type IPD in the 5–14 year age group decreased by 89% (95% CI 0–99), from 9 cases per 100 000 person-years to 1 per 100 000 person-years.

The estimates of PCV impact on IPD were unchanged in stratified analyses excluding outpatients and cases detected only by lung aspiration (appendix pp 27–28). When cases due to serotypes 1 or 5 were excluded, vaccine impact against PCV13 serotype disease was unchanged (appendix p 29). There was no change in the adjusted incidence of the control condition of non-pneumococcal bacteraemia between the baseline and 2016–17 period in the 2–59 month age group, adjusted IRR 0·88 (95% CI 0·64–1·21), nor in the 5–14 year age group, adjusted IRR 1·16 (95% CI 0·34–3·91; appendix p 30, pp 14–15).

PCV7 showed some impact on IPD in the 2–59 month age group from 2011 onwards with reduced cases due to serotypes 6A and 14 (appendix p 16). Following the introduction of PCV13 in 2011, cases of serotype 5 declined in 2012 while serotype 1 cases continued to be detected until 2014 (appendix p 16). However, by 2016–17 the incidence of serotype 1 IPD had declined by 94% (95% CI 53–99), from 22 cases to 1 case per 100 000 person-years. We detected zero cases of IPD due to PCV13 serotypes in 2016–17 in the 2–11 month age group (appendix p 18). 15 different serotypes contributed to a peak of non-vaccine type IPD in 2014; such cases then declined (appendix p 17).

In the 5–14 year age group there was a peak of serotype 5 IPD in 2010 with zero cases in 2016–17 (appendix p 17). Serotype 1 cases persisted for a greater period but became rare in 2016–17. There were eight cases of PCV13-type IPD in the 5–14 year age group in the first 2 years of surveillance (all serotype 1 or 5) and one case in 2016–17 (aIRR 0·11, 95% CI 0·01–1·00). Across all age groups, there were 27 episodes of vaccine failure: three for serotypes 1 and 19A, five for serotype 23F, and eight for serotype 14 (appendix p 31).

Following the introduction of PCV7 and PCV13 the incidence of radiological pneumonia initially fell, however a peak then occurred in 2015 in the 2–23 month age group. Incidence in all age groups then declined (figure 2). The adjusted incidence of radiological pneumonia in the 2–59 month age group decreased from 10·5 to 7·0 per 1000 person-years, a 33% reduction (95% CI 24 to 40). Reductions in radiological pneumonia were greatest in the 2–4 year age group (table 4). The adjusted reduction in radiological pneumonia in the 5–14 year age group was 27% (95% CI −5 to 49), from 0·7 per 1000 person-years to 0·5 per 1000 person-years (table 4, appendix p 19).

Compared to our earlier findings,^6^ we observed greater impact on the following pneumonia endpoints. The adjusted incidence of clinical pneumonia in the 2–59 month age group declined by 5% (95% CI 1–9), from 68·8 per 1000 person-years to 65·1 per 1000 person-years (table 4). Hospitalised clinical pneumonia fell by 27% (95% CI 22–31), from 43·1 per 1000 person-years to 31·5 per 1000 person-years, with the greatest absolute reduction being in the 2–11 month age group, with a fall from 105·3 per 1000 person-years to 79·1 per 1000 person-years, a 25% decline (95% CI 18 to 31).

There was a 60% (95% CI 51–68) decline in the adjusted incidence of hypoxic pneumonia in the 2–59 month age group, from 4·7 per 1000 person-years to 1·9 per 1000 person-years. *S pneumoniae* was isolated from 168 (21·0%) of 801 cases of hypoxic pneumonia and 337 (1·5%) of 21 954 clinical pneumonia cases. The adjusted incidence of the control condition of non-pneumococcal bacterial pneumonia was not significantly different in the baseline and 2016–17 periods (appendix p 36). The adjusted incidence of bronchiolitis declined by 31% (95% CI 22–39) in the 2–11 month age group and 21% (95% CI 6–33) in 12–23 month age group (appendix p 36).

The prevalence of malaria in patients across all age groups fluctuated between 6% and 19% between 2008 and 2016 and fell to 3% in 2017 (appendix p 20). The prevalence of malnutrition and blood culture contamination was stable throughout (appendix pp 21–22).

## Discussion

Using population-based surveillance over 10 years, we found large reductions in IPD and moderate reductions in pneumonia incidence in young children after introduction of PCV13 using the standard three-dose schedule without booster or catch-up dose. Notably, in the 2–11 month age group we observed zero vaccine-type cases in 2016–17. This is the first population-based study of the long-term impact of the introduction of PCV with the approach that is employed in almost all low-income countries.

The introduction of PCV in high-income countries has been associated with large reductions in IPD^12^, ^13^, ^14^ and substantial falls in pneumonia admissions.^15^, ^16^, ^17^ Early studies of the impact of PCV13 in South Africa^3^ and The Gambia^5^, ^6^ reported moderate effects but longer-term observation has been needed to determine the full programmatic impact. A recent series of papers examining data from the WHO-coordinated Paediatric Bacterial Meningitis Surveillance in a number of African countries indicates early changes in meningitis and pneumonia epidemiology associated with the introduction of PCV, but calculations of incidence before and after introduction were not available.^18^ In Basse, 8 years after the introduction of PCV, we observed a 92% reduction in PCV13-type IPD in the 2–59 month age group, and elimination of vaccine-type IPD in the 2–11 month age group. These findings are reassuring as our earlier analysis showed persisting PCV13-type IPD in young children.^5^ Similar observations have been made in other settings.^12^, ^13^, ^14^ In coastal Kenya, 5 years after the introduction of PCV10 with catch-up vaccination to 5 years of age, vaccine-type IPD in the under-5 year age group declined by 92% and all IPD by 68%.^4^ The difference between our 91% reduction in PCV13-type IPD in the 2–23 month age group and the documented 57% reduction in South Africa seems to be primarily a result of the 8 years of vaccine use in The Gambia versus only 1 year at the time of the South African analysis.^3^

We did not observe significant serotype replacement disease. Secular trends might have contributed to this finding and study power was limited by the number of cases. Similar observations of limited serotype replacement in children have been made in some settings^4^, ^12^ with significant replacement disease in others.^18^, ^19^ Serotype replacement remains a concern, particularly in older age groups in high-income countries.^19^

The greater impact seen after 8 years of PCV introduction compared to our earlier estimates shows that programme impact continued to increase, even after 4 years of vaccine introduction. The 80% reduction in all IPD in the 2–59 month age group was greater than our previously reported 55% decline after 4 years of PCV7 or PCV13 introduction. This contrasts with the rapid impact of PCV10 introduction with catch-up immunisation to age 5 years in Kilifi, Kenya, where vaccine-type IPD in young children declined to zero after only 2 years.^4^ In Basse, maximal programmatic impact in the under-5 year age group was achieved 8 years after vaccine introduction. These findings argue for the use of catch-up programmes as is now recommended by WHO^20^ and supported by Gavi.^21^

Our finding of an 89% reduction in PCV13-type IPD in 5–14 year olds is similar to findings in Kenya where a 92% reduction in PCV10-type IPD was observed.^4^ We also found reductions in all IPD and radiological pneumonia in the 5–14 year age group, although the reductions were not statistically significant. Investigators in Kenya did observe significant reductions in all IPD in this age group^12^ but declines in radiological pneumonia were not significant.^16^ The power of our study to detect vaccine impact in older children was limited by small numbers of cases and further surveillance is needed to confirm the indirect impact of the standard schedule in settings of high transmission, such as the African meningitis belt.

In contrast to our previous analysis we are now able to confirm PCV13 impact against serotype 1, which is an important cause of disease in low-income settings. Similar impact has been reported elsewhere.^3^, ^13^, ^14^ Our observation is important as it supports the argument that serotype 1 disease can be prevented in this setting without requiring a booster dose.^22^

Even though our finding of a 33% decline in radiological pneumonia in the 2–59 month age group was greater than the previously reported 24% decline, it is lower than the 47% reduction observed in Israel^16^ and 48% reduction in Kenya.^23^ Given that the routine use of PCV is associated with indirect effects in addition to direct protection, and our use of a PCV against 13 serotypes, we expected the reduction in radiological pneumonia to be greater than the 37% efficacy measured in the Gambian PCV9 trial. A temporal increase in the PCV13 period in the proportion of children presenting with respiratory symptoms who have radiological pneumonia might explain this observation. Such a change may have been related to a temporal increase in respiratory viral disease.

The greater period of observation in this analysis allowed us to document a 5% decline in all clinical pneumonia in the 2–59 month age group, which was not evident in our previous report, and a 27% reduction in hospitalised clinical pneumonia (previously reported 8% reduction), we could also confirm a substantial 60% decline in hypoxic pneumonia (previously reported 65% reduction). The similarity to previous estimates against hypoxic pneumonia is likely a result of similar high coverage of PCV13 in the important 2–23 month age group during the period covered by our previous report. Indeed, our study supports the usefulness of hypoxic clinical pneumonia as an endpoint to assess pneumonia vaccines, especially in settings where radiology is not reliably available. The relatively low impact against clinical pneumonia is consistent with the PCV9 efficacy trial.^24^ The reason for this is unclear and may include the overlap of symptoms and signs of pneumonia with other diseases. Given the high burden of mortality associated with clinical pneumonia these children warrant further research.

Our study has several strengths. All screening, clinical investigation, case definitions, and laboratory practices were standardised and consistently applied throughout.^7^ The very low proportion of infants who received substantially delayed second or third doses of PCV confirms that our results relate to the use of a three-dose schedule without booster or catch-up dose. However, there are some limitations. There were only 16 months of surveillance before the introduction of PCV7 and we specified a 2-year baseline period, based on the low proportion of children who had received at least two doses for several months after vaccine introduction. Before-and-after studies are prone to bias and confounding due to changes in factors apart from vaccination. Our analysis provided some reassurance in this regard, with stable incidence of the control condition of non-pneumococcal bacteraemia, and no change in estimates in stratified analyses.

The substantial vaccine impact that we observed in The Gambia resulted from a PCV programme using a standard schedule of three primary doses without catch-up immunisation, the approach used in almost all low-income countries. Thus, given our findings, and the similar distributions of IPD serotypes elsewhere in Africa,^3^, ^4^ and in South Asia,^25^ our data have important implications for EPI programmes globally. Programmes that introduce PCV with reasonable coverage and timeliness can expect substantial reductions in pneumococcal disease and severe pneumonia; countries should consider catch-up vaccination when introducing PCV; and continued surveillance is needed to monitor for serotype replacement and determine whether indirect effects in older age groups will develop over a longer period of time. Our data will provide valuable information to countries that are yet to introduce PCV and those which have introduced it but are considering whether to maintain vaccination when they transition from Gavi financial support.

## Data sharing

Data collected for the study, including individual, deidentified participant data, and a data dictionary defining each field in the dataset, may be made available to others. Access will be granted following approval of an application for research analysis to the Gambian Government/MRC Joint Ethics Committee. The statistical analysis plan and informed consent forms will be made available upon request.

## Declaration of interests

RA was employed by GlaxoSmithKline Vaccines and received grant awards from WHO, Gavi, and the Bill & Melinda Gates Foundation whilst employed at MRC Gambia. MK, SH, and BG received grants from the Bill & Melinda Gates Foundation. MK received grants from Gavi, Merck, and Pfizer, and personal fees from Pfizer. All other authors declare no competing interest.
