# Supplementary material for: Impact of the introduction of pneumococcal conjugate vaccination on invasive pneumococcal disease and pneumonia in The Gambia: 10 years of population-based surveillance
Source: Lancet Infect Dis. 2021 Sep;21(9):1293–302. doi: 10.1016/S1473-3099(20)30880-X (PMC8384632; doi:10.1016/S1473-3099(20)30880-X)
Supplement: Supplementary appendix [file mmc1.pdf]

THE LANCET
Infectious Diseases

Supplementary appendix

This appendix formed part of the original submission and has been peer reviewed. We post it as supplied by the authors.

Supplement to: The Gambia Pneumococcal Surveillance Group, Mackenzie GA, Hill PC, Jeffries DJ, et al. Impact of the introduction of pneumococcal conjugate vaccination on invasive pneumococcal disease and pneumonia in The Gambia: 10 years of population-based surveillanc. *Lancet Infect Dis* 2021; published online July 16. https://doi.org/10.1016/S1473-3099(20)30880-X.

Supplementary Web Appendix

Impact of the introduction of pneumococcal conjugate vaccination on invasive pneumococcal disease and pneumonia in The Gambia: 10 years of population-based surveillance

1. SUPPLEMENTARY METHODS

1.1 Study objective

The objective of this study was to determine the impact of the routine introduction of pneumococcal conjugate vaccine (PCV) on the incidence of radiological pneumonia and invasive pneumococcal disease in rural Gambia, at the population-level.

1.2 Study setting

Clinical surveillance was conducted at all the health facilities which provide inpatient services in the area of the Basse Health and Demographic Surveillance System (BHDSS) in the Upper River Region, on the south bank of the River Gambia. The health needs of this population are served by the Basse Health Centre, which receives referrals from five outlying health facilities, Gambisara, Demba Kunda, Fatoto, Garawol, and Koina. A sixth outlying facility began operation in Sabi in February 2016 and surveillance was initiated there on September 1st, 2016. There are no private health facilities in this area which provide inpatient services. Maternal-child-health teams provided immunisation services at three base clinics and 37 outreach sites. In 2009, the estimated infant and under-5 year mortality rates in the BHDSS were 33 and 62 per 1,000 live births respectively. HIV prevalence among antenatal women in the area is around 1.0%.[1] *Plasmodium falciparum* is endemic with seasonal transmission during the wet season between August and December each year. The majority of the population engage in subsistence farming.

1.3 Surveillance population

The BHDSS, which covers an area of 1,111 square kilometres, has been in operation since 2007. All residents of the BHDSS aged 2 months and greater were included in the study. Residence was defined as continual residence within the BHDSS for 4 or more months, or birth to a female resident, as determined at 4-monthly visits to all households. The population of the BHDSS is enumerated every 4 months with recording of all births, deaths, and migrations. The estimated population in 2008 was 146 876, increasing to 181,740 in 2017. Although individuals who are not resident in the BHDSS do present to the health facilities in the area, this analysis includes only individuals who were confirmed resident at the time of illness. Residence was confirmed by the individual having been enumerated in the BHDSS population listing. Individuals enrolled in surveillance who were not found in the BHDSS population listing were visited at their household in order to ascertain residential status; if confirmed as residents they were enumerated through BHDSS procedures. This analysis is restricted to the age group 2 months to 14 years.

1.4 Case ascertainment

Patients were eligible for recruitment if they presented as an outpatient, or were admitted to one of the health facilities in the BHDSS between May 12, 2008 and December 31, 2017. Patients who presented to any of these health facilities were screened for referral to a clinician by designated surveillance staff using standardised criteria (Table S1). Clinicians assessed patients referred to them using standardised diagnostic criteria (Table S2). A diagnosis of suspected pneumonia, septicaemia, and/or meningitis led to standardised investigations (Table S3).

Chest radiographs were taken on all cases of suspected pneumonia and if the attending clinician judged the investigation beneficial to patient management.

Clinicians recorded findings of clinical history and examination of every patient on standardised forms. For those aged 2-59 months, the surveillance diagnosis of suspected pneumonia was based on the clinician's clinical judgement or a presenting complaint of cough or difficulty breathing for less than 14 days and the presence of one or more of the following criteria: raised respiratory rate for age (>50 breaths/min for children aged 2-11 months, >40 breaths/min for children aged 12-59 months), lower chest wall in-drawing, nasal flaring or grunting, peripheral oxygen saturation less than 92%, or focal chest signs (dull percussion note, coarse crackles, or bronchial breathing). During the course of the project it became apparent that there was a group of children who presented repeatedly with an illness that met criteria for undertaking a chest X-ray and blood culture, but for whom, the clinicians' clinical diagnosis was one of reactive airways disease. After repeated presentations of a child for whom a diagnosis of reactive airways disease had been made, clinicians exercised their judgment regarding requests for further X-rays and blood cultures.

Weight was recorded using a digital scale (TANITA, Arlington Heights, USA) and height using a ShorrBoard® (Weigh and Measure, Olney, USA). Peripheral O_2 saturation (Nellcor N-65, Covidien, Colorado) was recorded routinely for all enrolled patients. Rapid malaria tests (ICT Diagnostics, Cape Town) were performed for all patients during the malaria transmission season from August to December each year.

Blood cultures were performed for all cases of suspected pneumonia, sepsis, or meningitis. Lumbar puncture was undertaken in cases of suspected meningitis. Lung aspiration was undertaken in selected cases of pneumonia when the following criteria were met: a) a large radiographical area of dense, peripheral, pneumonic consolidation, b) stable respiratory status, and c) written informed consent provided by the patient or parent or guardian of the participant child. Pleural fluid was aspirated for selected patients if a large pleural effusion was demonstrated on chest radiograph.

Radiographs were obtained using a portable system (HF-110A, DynaRad, Illinois, USA) with a consistent radiographical technique and digital processing (CR 120/140, Kodak, New York, USA). Procedures to produce digital images were in accordance with WHO recommendations.[2] Two readings of each radiograph (masked to identity and date) were undertaken by three independent readers and readings discordant for end-point consolidation were resolved by a paediatric radiologist. All readers were calibrated to the WHO standard for radiological pneumonia with consolidation.[2] All readers were required to achieve very high levels of agreement on end-point consolidation with blinded samples of the WHO standard set of 222 radiographs (kappa statistic >0.8) before they read the study radiographs.

Blood was collected for culture using a sterile technique and inoculated into a culture bottle (Bactec Peds Plus or conventional tryptone soy and brain heart infusion bottles for a minority of samples collected at night in outlying clinics). The weight of blood culture bottles was measured before and after sample collection. Lung aspirates were transported immediately to the MRC Basse laboratory and inoculated onto agar and examined using Gram stain. Cerebrospinal fluid, pleural fluid, and other microbiological samples were processed consistently using standard methods.[3]

1.5 Case definitions

All events included in the analysis of longitudinal data were restricted to residents of the BHDSS. IPD was defined as an illness clinically compatible with this diagnosis accompanied by isolation of *S. pneumoniae* from a normally sterile site. We classified IPD as being caused by a) PCV13 vaccine serotypes: 1, 3, 4, 5, 6A, 6B, 7F, 9V, 14, 18C, 19A, 19F, and 23F or b) non-PCV13 vaccine serotypes being all other types. Non-typeable isolates were excluded. Episodes were considered as separate events if the first and subsequent consultations were at least 30 days apart, or if pneumococcus of a different serotype was isolated during each episode.

Events were defined as radiological pneumonia if a patient had a suspected diagnosis of pneumonia and the radiological end-point consolidation, as per the procedure for classification according to the WHO standard.[2] If multiple radiographs were taken during an episode of illness the worst radiographical appearance in the 3 days following the date of screening was accepted at final.

Clinical pneumonia was defined as cough or difficulty breathing for <14 days accompanied by: raised respiratory for age, lower chest wall in-drawing, nasal flaring, grunting, O_2 saturation <92%, altered consciousness, inability to sit or feed, convulsions, dull chest percussion note, coarse crackles, or bronchial breathing, with isolation of *S. pneumoniae* from a sterile site.

Hypoxic pneumonia was defined as clinical pneumonia with peripheral O_2 saturation <90%. Bronchiolitis was defined as clinical pneumonia with wheeze on chest auscultation without bronchial breathing or dullness to percussion combined with an absence of end-point consolidation on chest radiograph.

Vaccine failure was defined as IPD caused by a PCV7 vaccine serotype after administration of two or more doses of PCV7, or IPD caused by a PCV13 vaccine serotype after administration of two or more doses of PCV13, with the event occurring greater than 14 days after the second dose of vaccine.

1.6 Laboratory methods

An automated system (Bactec 9050, Becton Dickinson, UK) was used for blood cultures. Bottles that signalled positive were sub-cultured onto blood agar, chocolate agar, and McConkey agar. Bottles which failed to signal within 5 days were considered negative. Isolates grown on these sub-cultures were identified using conventional microbiological techniques and biochemical tests (API, Biomerieux). Other sterile site samples were processed using consistent and standardised techniques.[3] *S. pneumoniae* was identified by colony morphology, susceptibility to ethylhydrocupreine and, if susceptibility was equivocal, by bile solubility, and reaction with polyvalent antisera (Statens Serum Institut, Copenhagen, Denmark). Isolates classified as contaminants included coagulase-negative *Staphylococcus*, *Bacillus* species, *Micrococcus* species, and *Streptococcus viridans*.

Pneumococcal isolates were transported to the MRC Fajara laboratory for serotyping using a latex agglutination assay which employs factor and group-specific antisera (Statens Serum Institut, Copenhagen, Denmark). Serotypes 6A and 6B were differentiated from 6C by polymerase chain reaction using a 6C-specific DNA primer (CDC, Atlanta, USA). External quality control serotyping was performed at the National Institute for Infectious Diseases in South Africa for 10% of isolates and for all isolates of serogroups 6 or 9. The laboratories in Basse and Fajara submitted to external quality assurance programmes throughout the study (UK National External Quality Assessment Service and OneWorld Accuracy International, Canada).

1.7 Vaccination

All vaccines were delivered by the Gambia Government EPI. PCV7 was introduced on August 19, 2009 without a catch-up programme and PCV13 was introduced in May 2011. The schedule included three doses at ages 2, 3, and 4 months. At the time of the introduction of PCV7 in 2009, children aged <6 months were eligible to receive three doses, while older children were eligible for one dose. Surveillance staff attended all vaccination clinics and recorded the administration of all vaccine doses in the BHDSS using a real-time electronic system.

In order to illustrate the rate of uptake of PCV in different age groups included in the analysis, we plotted the coverage over time of two or more doses of PCV7 and PCV13 in the 2-11 month, 12-23 month, 2-4 year, and 5-14 year age groups. These age specific coverage estimates over time were calculated using individual level records from the BHDSS with coverage calculated by age-strata on each day of the study period and plotted (figure S2). To demonstrate the timeliness of vaccination, we calculated the coverage of two doses before 4, 5, and 6 months of age and three doses before 5, 6, and 7 months of age in the cohort of BHDSS residents born in 2012-2017.

1.8 Population denominator

Annual incidence was calculated using BHDSS estimates of the age-specific mid-point population in each year. Comparisons of before and after incidence used weighted averages of the mid-point populations in the respective age-strata and annual time periods, May 12, 2008 – May 11, 2010 and 2016/17.

1.9 Extrapolation of case counts in 2008 and 2010

We extrapolated the number of unobserved cases between January 1 and May 11, 2008 and also during the flood period between October 5 and November 3, 2010; radiological pneumonia surveillance was interrupted for 8 days between October 5 and 12, 2010. These extrapolated cases were used for plots of annual incidence over time but not used in calculations of incidence rate ratios.

The expected number of cases from January 1 to May 11, 2008 was the product of the number of cases between May 12 and December 31, 2008 and the average of the ratios of annual cases occurring during January 1 to May 11 compared to May 12 to December 31 in 2009 and 2011-2017. The age and serotype distribution of the extrapolated cases was the same as the age and serotype distribution of observed cases in 2008 and 2009.

Unobserved cases of radiological pneumonia in 2010 were extrapolated for the eight days of interrupted pneumonia surveillance during the flood period. Pneumococcal pneumonia cases were extrapolated for the 30 days of interrupted IPD surveillance. The expected number of unobserved radiological and pneumococcal pneumonia cases respectively was the product of the number of observed cases in 2010 and the average of the annual ratios of cases which occurred between October 5–12, and October 5-November 3, in 2009, and 2011-2017. The age and serotype distribution of the extrapolated cases was the same as for the observed cases in 2010.

1.10 Adjustment of annual case counts

The number of children presenting to health facilities and screened for referral to surveillance clinicians per unit population increased over time between 2008 and 2015. We assumed that this increase was largely due to increasing awareness of the presence of an improved health system. The rate of patient presentation to the surveillance system then decreased from 2015 to 2017.

In our earlier analyses,[4, 5] we corrected the annual counts of events using an approach which has been used by a number of other investigators.[6, 7] We corrected for these changes in the sensitivity of case ascertainment by adjusting the annual counts of IPD and pneumonia cases, by age group, and assuming the serotype distribution was the same as that of the observed cases each year. As in our earlier analyses, annual, age-specific case counts were adjusted using the difference between the mean rates of: a) referral of patients to surveillance clinicians (for pneumonia endpoints) and b) enrolment of patients eligible for investigation (for IPD endpoints) during the study period and non-linear regression curves fitted to plots of these annual rates over time. The peak rate of presentation and referral of children occurred in 2014 and 2015, while the rate of presentation to the surveillance system fell from 2015 to 2017. Thus, our adjustment increased the number of cases in the early and later years of surveillance with reduced numbers of cases in 2014 and 2015. The rationale for the adjustment was correction for a surveillance artefact of variable patient enrolment and testing, and potential bias due to variation in surveillance sensitivity over time, as this was not plausibly associated with the introduction of PCV.

Restating our approach in other words, we adjusted annual, age-specific counts of IPD to the mean rate of enrolment of patients eligible for investigation during the study period. We adjusted annual, age-specific counts of pneumonia to the mean rate of referral during the study period. Thus, interpretation of the adjusted analyses assumes a constant rate of enrolment of patients eligible for investigation and a constant rate of rate of referral to clinicians during the observation period. For consistency with our previous analyses, we present both crude and adjusted estimates in this analysis.

1.11 Surveillance time periods for the before and after analysis

The baseline period was from May 12, 2008 until May 11, 2010. The vaccine period was from January 1, 2016 until December 31, 2017.

Incidence was defined as the number of cases divided by the mid-point population at risk. Vaccine impact was calculated as the difference in age-specific incidence subtracting the incidence after vaccination from the incidence before the introduction of PCV,

$$IRD = Incidence\ before\ PCV - Incidence\ after\ 13PCV$$

The incidence rate ratio (IRR) was calculated as the ratio of incidence after to that before PCV, introduction indicating vaccine effectiveness at the population level. Vaccine effectiveness was calculated as (1-IRR) x 100.

$$IRR = Incidence\ after\ 13PCV / Incidence\ before\ PCV$$

The IRR analysis was based on a Poisson distribution. We assessed the validity of this distributional assumption by modelling the age-specific incidence of radiological pneumonia and IPD using pre-PCV data from 2008–2009. Our assessment of distributional validity indicated the Poisson distribution was acceptable for radiological pneumonia in the 2-11 month, 12-23 month, and 5-14 year age groups but that there was significant over-dispersion in the 2-4 year

age group. We therefore used a variance inflation factor of 1.219 to account for the over-dispersion in the 2–4 year age group. Our assessment of distributional validity indicated the Poisson distribution was acceptable for IPD in the 2-4 year age group but that there was significant over dispersion in the 2-23 month and 5-14 year age group. We therefore used variance inflation factors of 1.057 and 1.268 to account for the over-dispersion in the 2–23 month and 5-14 year age groups respectively.

1.12 Bias and confounding

To investigate potential bias due to temporal changes in health care seeking behaviour and case severity we conducted an *a priori* stratified analysis excluding outpatients. The IRR estimates using the crude data and data excluding outpatients were compared and if they differed by greater than 20% we concluded that clinically significant bias was present.[8] We conducted these stratified analyses of the IRR using crude data and data with adjusted case counts accounting for changes over time in the sensitivity of case ascertainment (section 4.10). We determined the adjusted, stratified, and age-specific annual case counts by multiplying the adjusted count by the proportion of radiological pneumonia cases treated as an outpatient in the baseline and PCV13 periods and by age strata.

In order to account for potential bias due to temporal changes in patient investigation and confounding due to secular trends in serotypes we conducted *a priori* stratified analyses excluding cases of pneumococcal pneumonia and IPD detected by lung aspiration alone and cases caused by serotype 1 or 5. We determined the adjusted, stratified, and age-specific annual case counts by multiplying the adjusted count by the proportion of cases detected by lung aspiration alone and the proportion due to serotype 1 or 5 in the baseline and PCV13 periods within age strata.

We evaluated potential bias due to temporal changes in the rate of patient presentation to the surveillance system by comparing the crude estimates of vaccine impact with estimates using adjusted annual case counts. We considered significant bias to be present if the adjusted compared to crude estimates differed by greater than 10%. If the adjusted and crude estimates differed by 10% or less we concluded that significant bias was not present.[8] If the adjusted and crude estimates differed by greater than 10% we concluded that significant bias was present.

To assess the effect of temporal trends related to bacterial pneumonia and IPD, we evaluated the crude and adjusted incidence of non-pneumococcal pneumonia and non-pneumococcal bacteraemia obtained by the surveillance system, as control conditions. Non-pneumococcal pneumonia was defined as a case of clinical pneumonia with a positive blood culture in which the isolate was not a contaminant, *S. pneumoniae*, *Serratia liquefaciens*, or *N. meningitidis*. *S. liquefaciens* was not included as we experienced a nosocomial epidemic of this bacterium in the latter half of 2010. Non-pneumococcal bacteraemia was defined as a positive blood culture in which the isolate was not a contaminant, *S. pneumoniae*, *Serratia liquefaciens*, or *N. meningitidis*. Meningococcal isolates were not included in non-pneumococcal pneumonia nor bacteraemia because of the epidemic nature of meningococcal disease; in fact, the surveillance area experienced an epidemic of meningococcal disease in 2012.[6] Non-pneumococcal pneumonia and non-pneumococcal bacteraemia were detected using the same procedures as for detection of pneumococcal pneumonia and bacteraemia and it is unlikely to be influenced by PCV. Therefore, the incidences of non-pneumococcal pneumonia and non-pneumococcal bacteraemia are robust control conditions indicating potential changes in the sensitivity of surveillance and/or changes in population risk factors for bacteraemia and bacterial pneumonia which are unrelated to PCV. Annual counts of non-pneumococcal pneumonia and non-pneumococcal bacteraemia were extrapolated in 2008 and 2010 in same manner as for radiological pneumonia and IPD respectively. We also adjusted the annual and comparison period counts of non-pneumococcal pneumonia and non-pneumococcal bacteraemia in order to evaluate the IRR.

In our setting, a significant risk factor for radiological pneumonia and IPD is malnutrition. Therefore, we also evaluated whether there were any changes over time in the prevalence of malnutrition in patients who were enrolled in surveillance with suspected pneumonia, septicaemia, or meningitis.

2. **SUPPLEMENTARY FIGURES**

Figure S1. Map showing the location of the Basse Health and Demographic Surveillance Systems in the rural east of The Gambia, West Africa

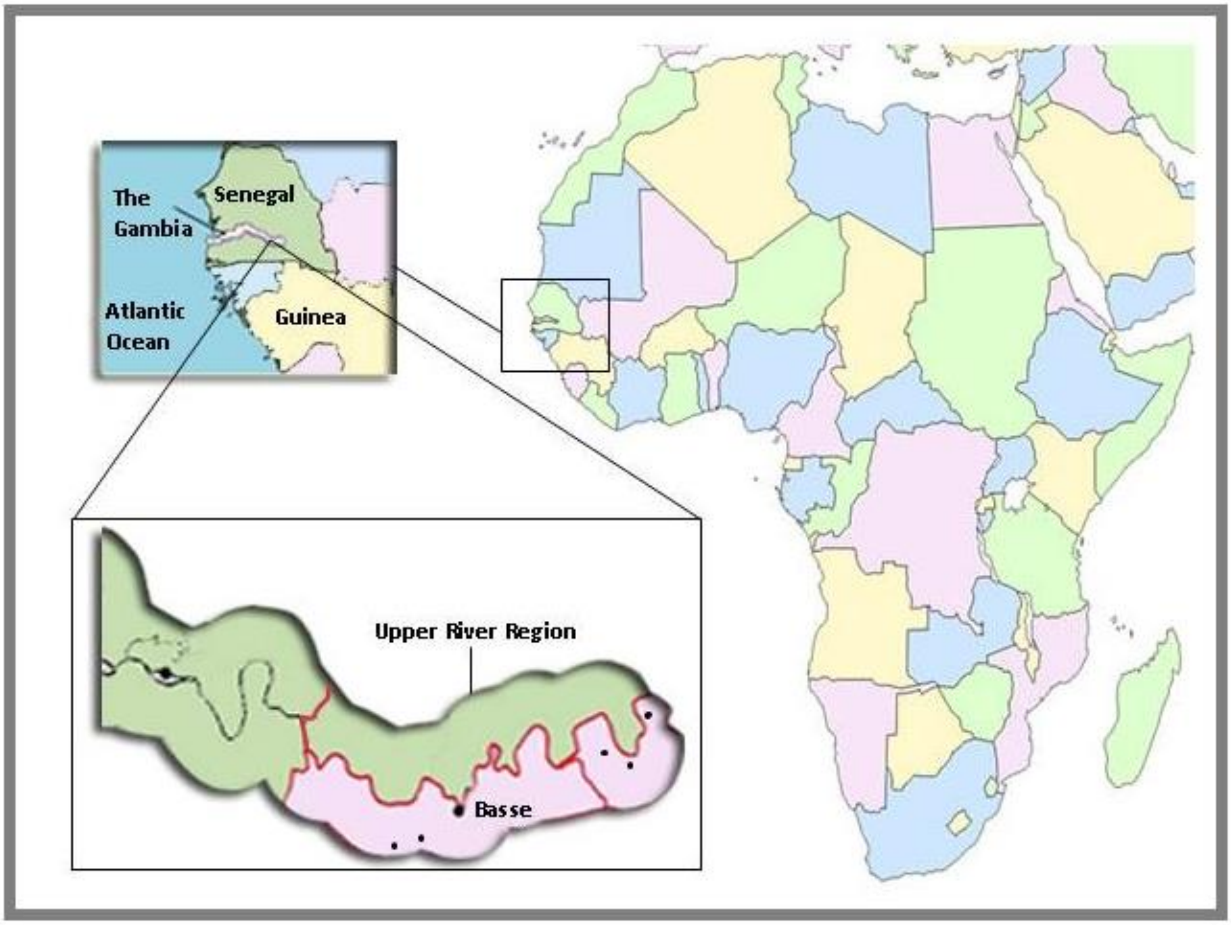

Figure S2. Coverage of two or more doses of A) PCV7 and B) PCV13, by age group over time

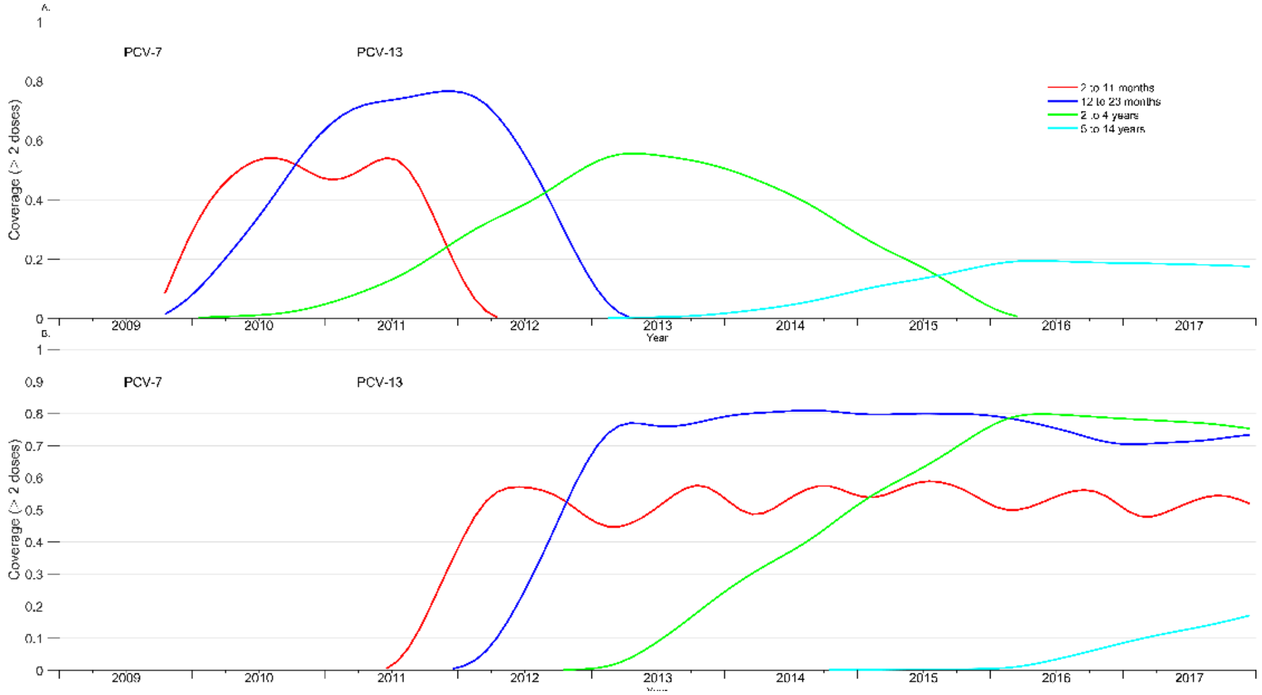

Figure S3. Proportion of patients aged 2 months to 14 years meeting referral criteria who were assessed by a clinician, by year of the study

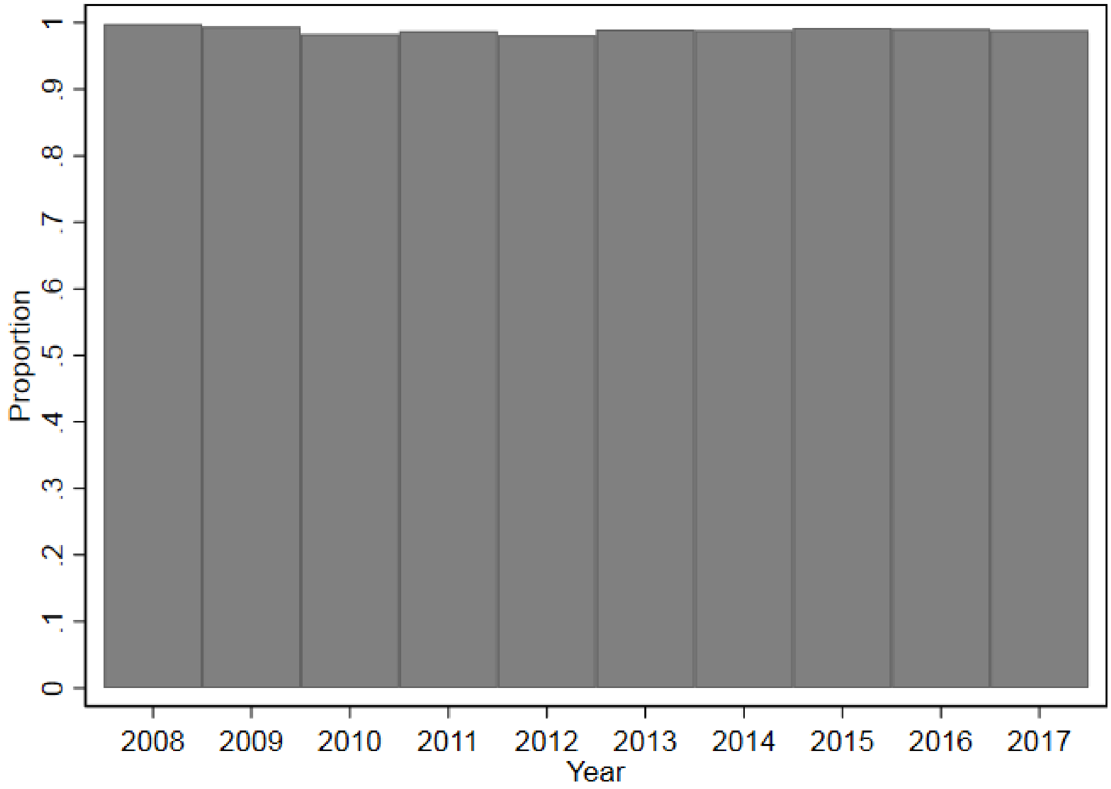

Figure S4. Proportion of patients aged 2 months to 14 years meeting referral criteria who had suspected pneumonia, septicaemia or meningitis, by year of the study

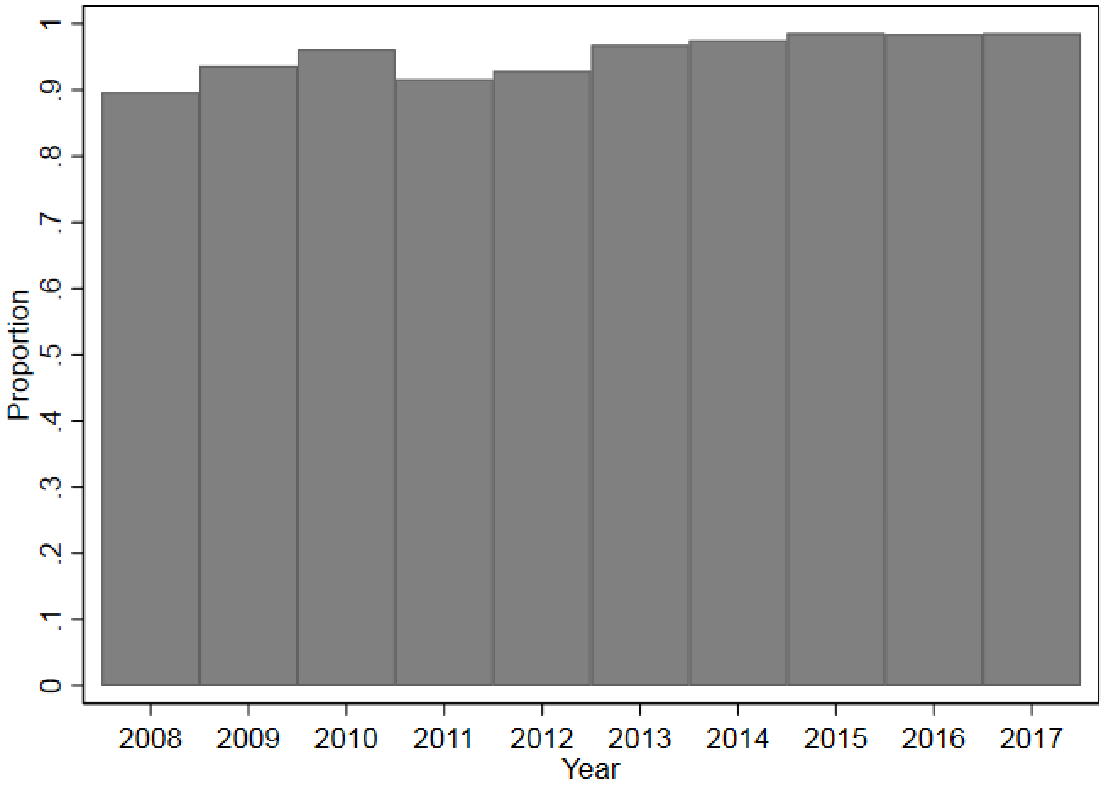

Figure S5. Proportion of patients aged 2 months to 14 years with suspected pneumonia, septicaemia or meningitis who had a microbiological investigation, by year of the study

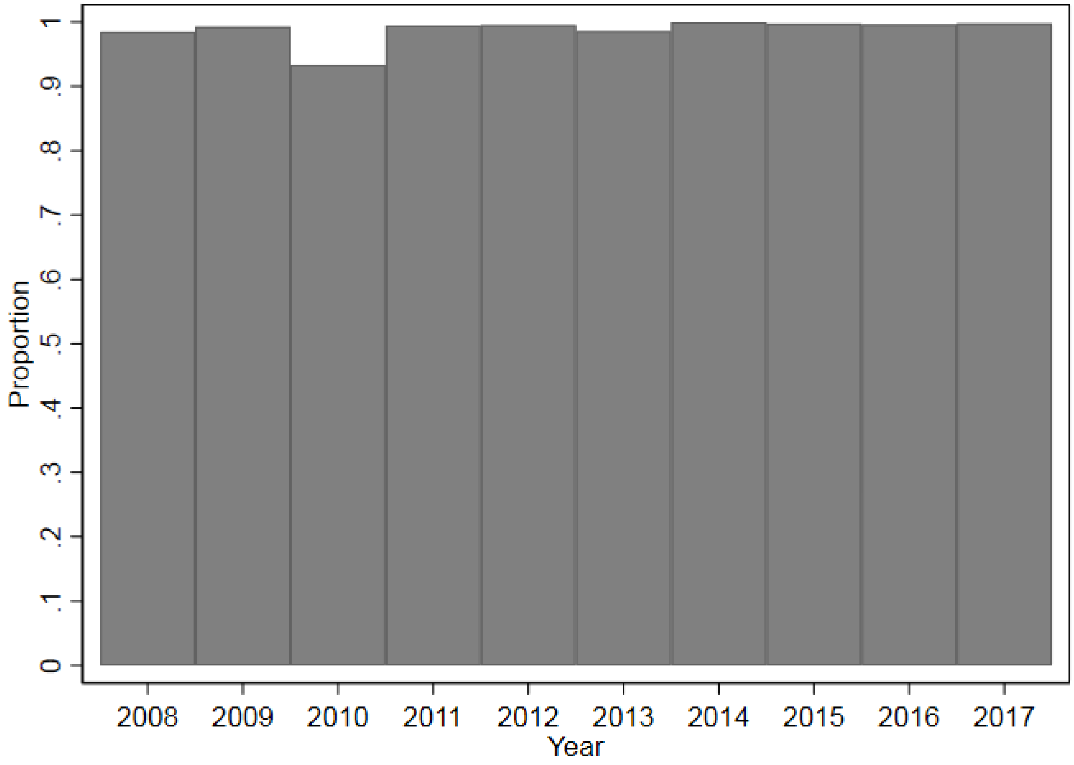

Figure S6. Proportion of patients aged 2 months to 14 years with suspected pneumonia who had a chest radiograph, by year of the study

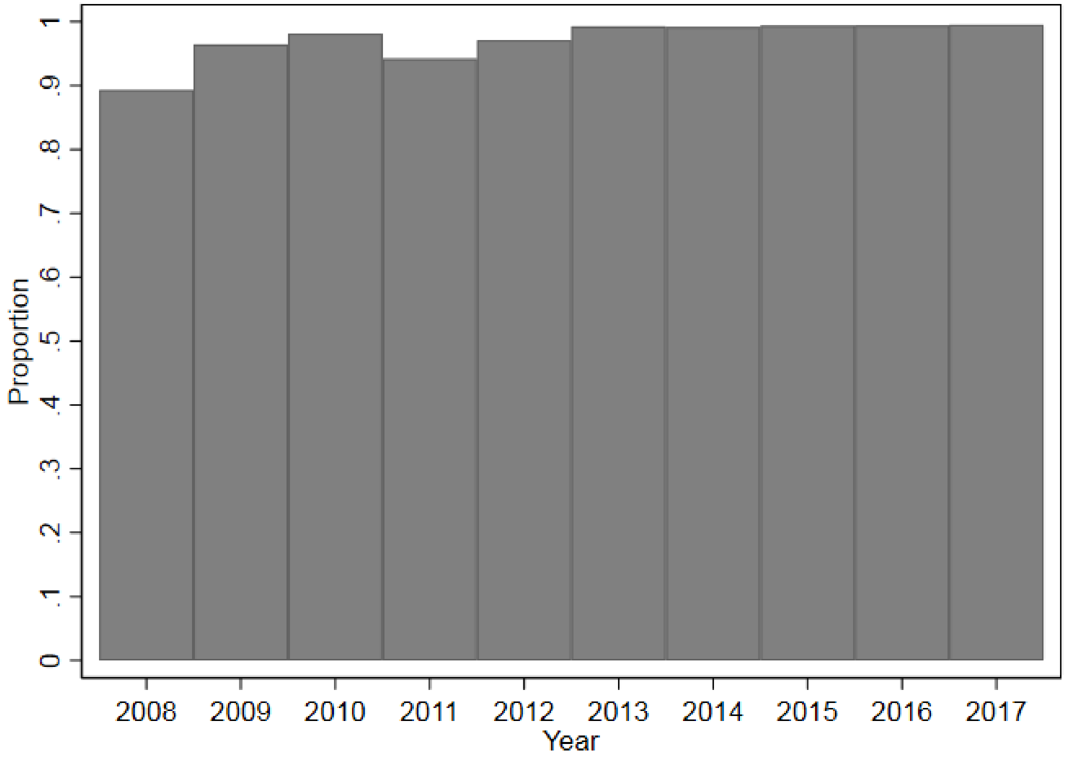

Figure S7. Proportion of patients aged 2 months to 14 years with suspected pneumonia who had a lung aspirate sample collected, by year of the study

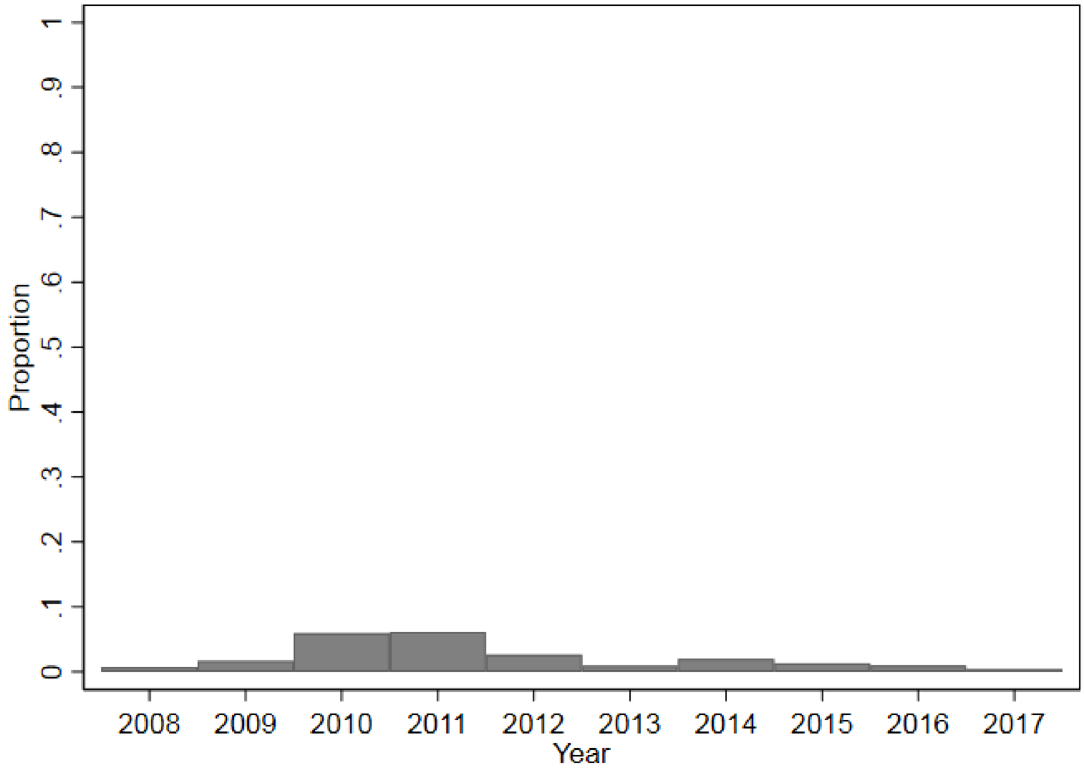

Figure S8. Adjusted annual incidence of invasive pneumococcal disease in children aged 5-14 years between 2008 and 2017

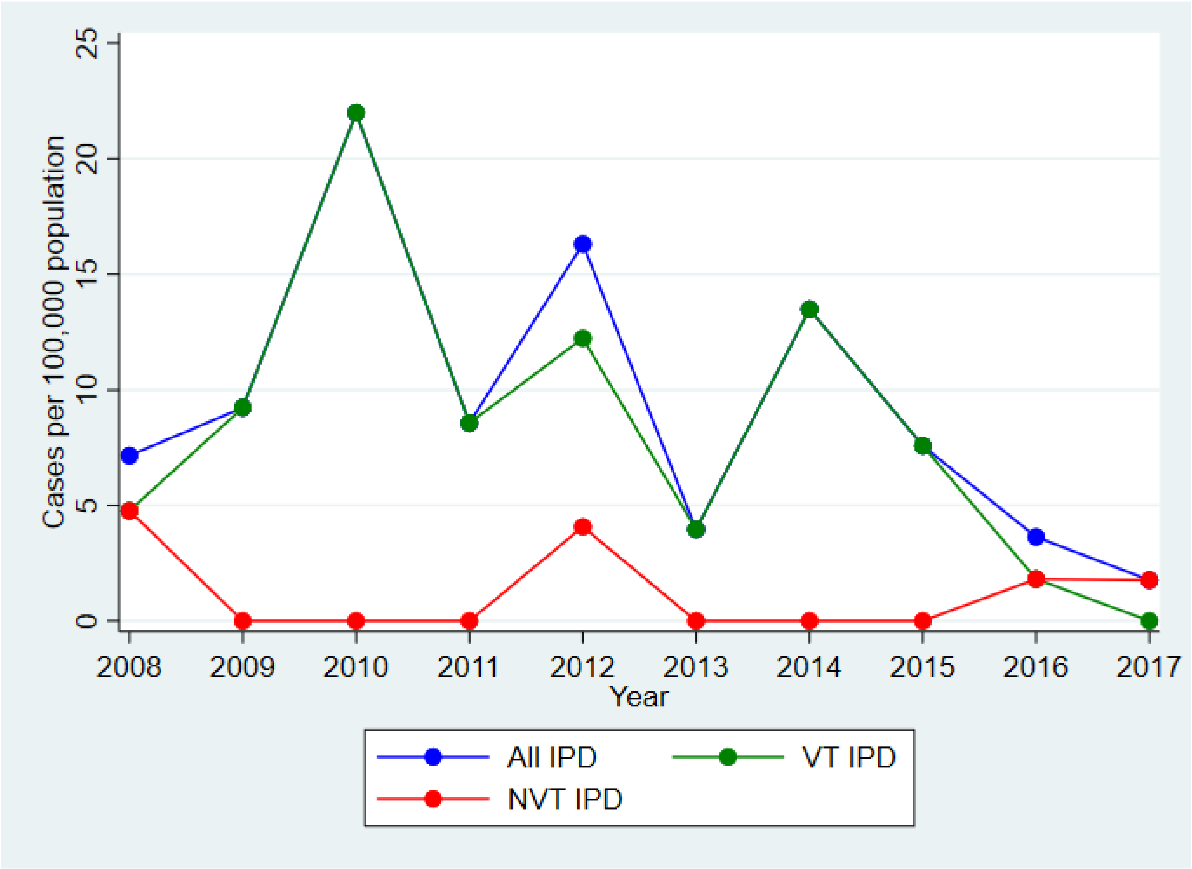

Figure S9. Adjusted annual incidence of non-pneumococcal bacteraemia in young children between 2008 and 2017

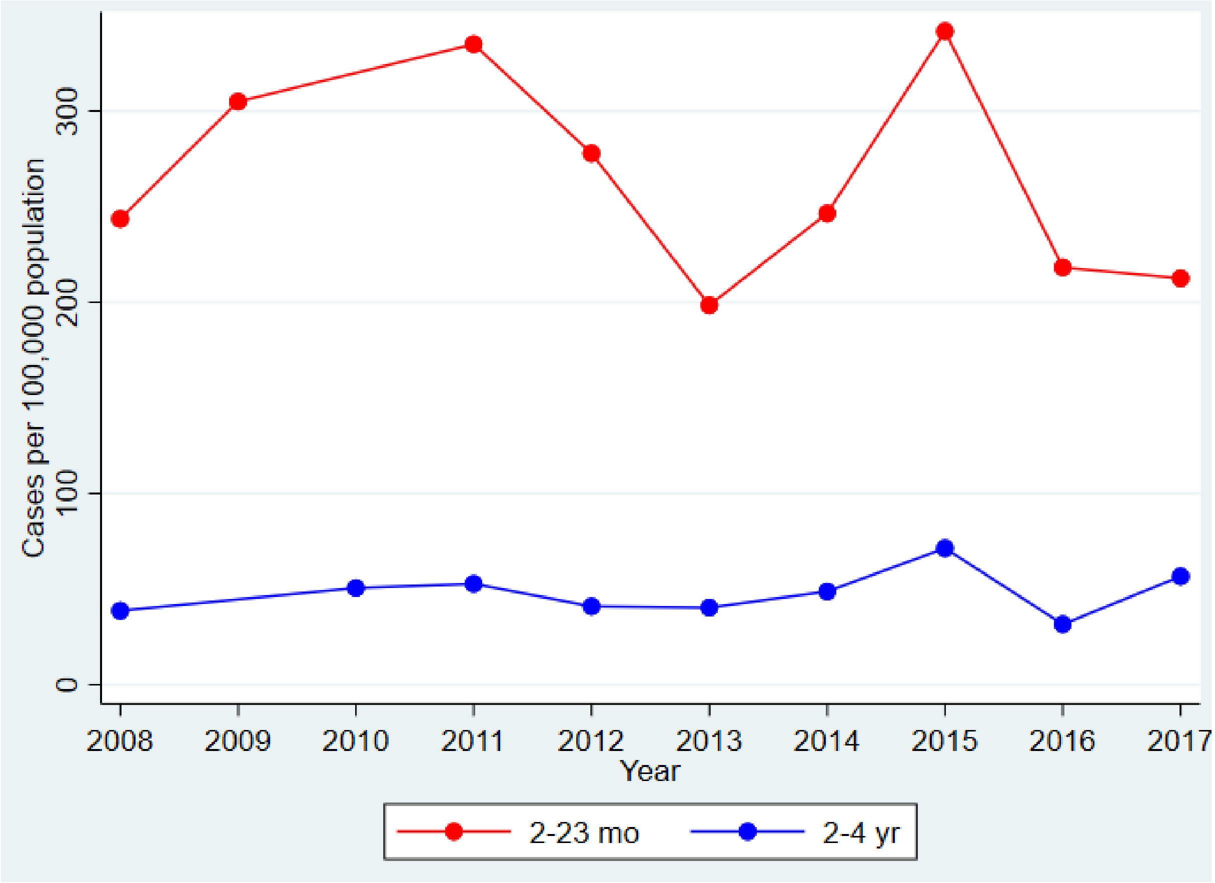

Figure S10. Adjusted annual incidence of non-pneumococcal bacteraemia in children aged 5-14 years between 2008 and 2017

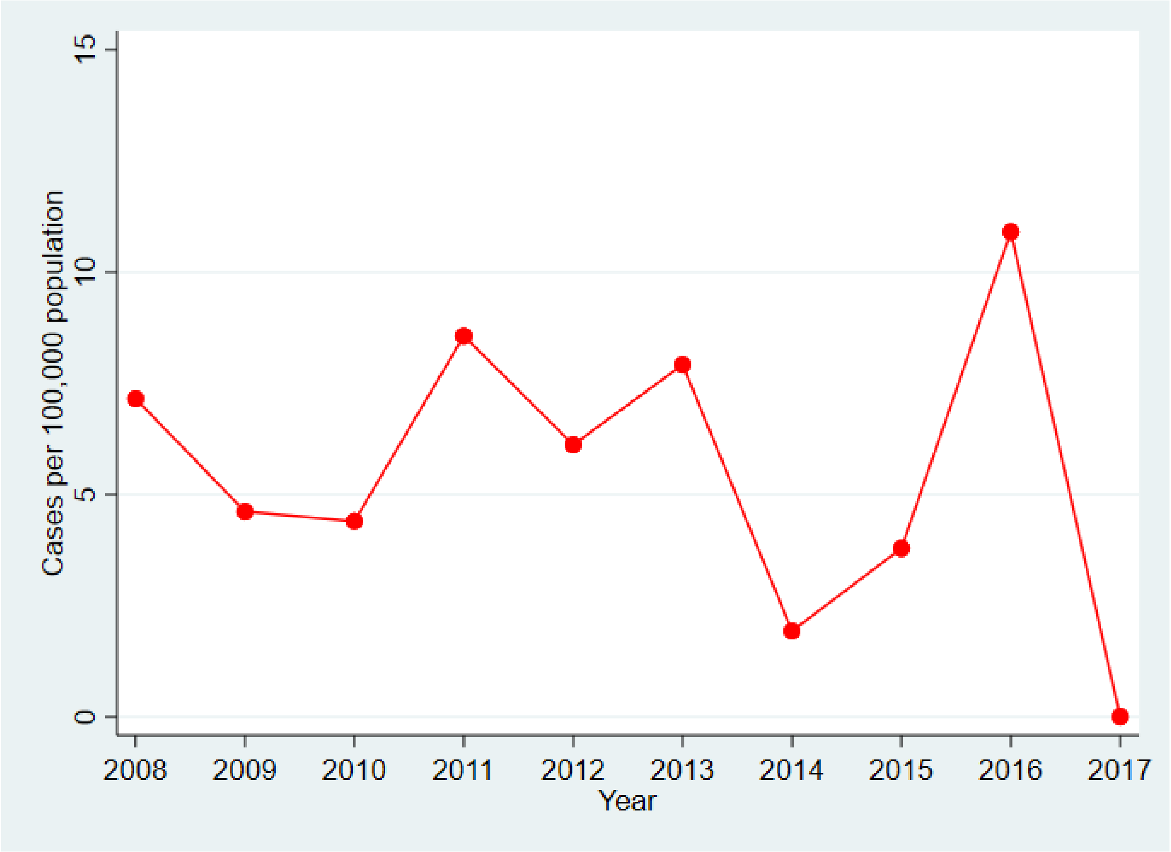

Figure S11. Annual counts of serotype-specific invasive pneumococcal disease; panels A – C refer to age 2–59 months and panel D to age 5-14 years; panel A - PCV7 serotypes, panel B - PCV13 only serotypes, panel C - non-PCV13 serotypes, and panel D - all serotypes

A

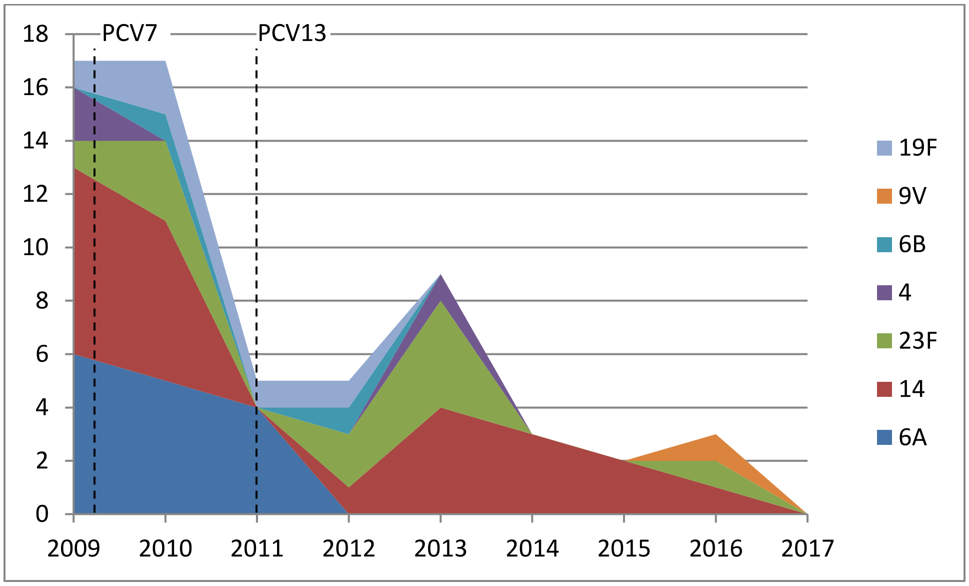

B

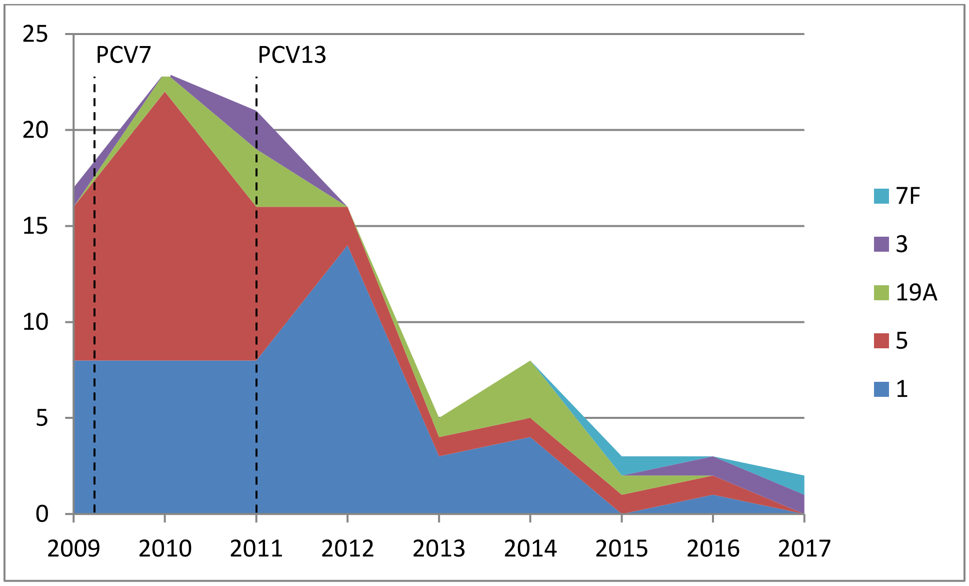

C

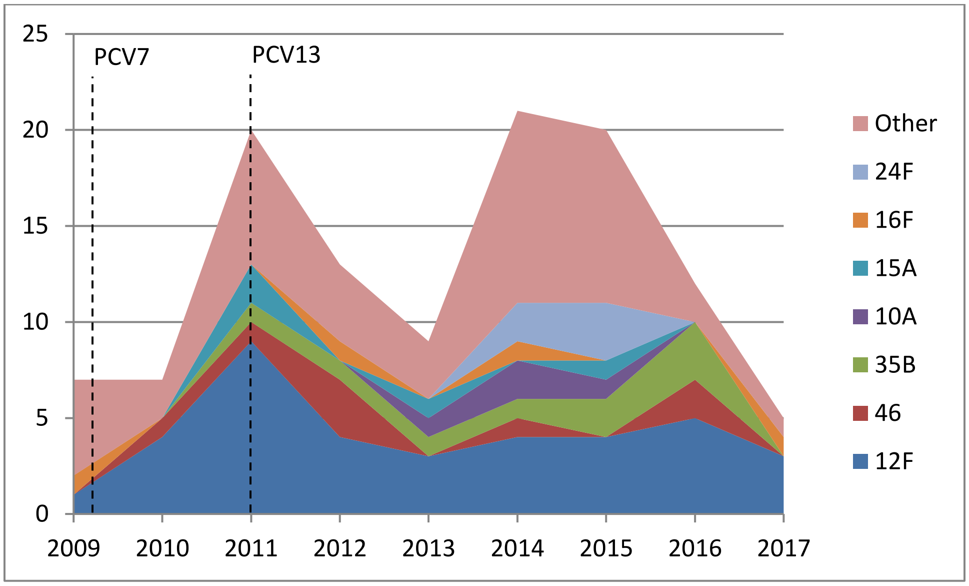

Serotypes shown separately if four or more episodes. Other serotypes (pink colour) with three episodes: 7C, 12B, 17F, 18A, 34; two episodes: 2, 8, 9A, 9N, 13, 24F, 25A, 25F, 33F; one episode: 10F, 11B, 12A, 15B, 18A, 20, 21, 22A, 23B, 33D, 40.

D

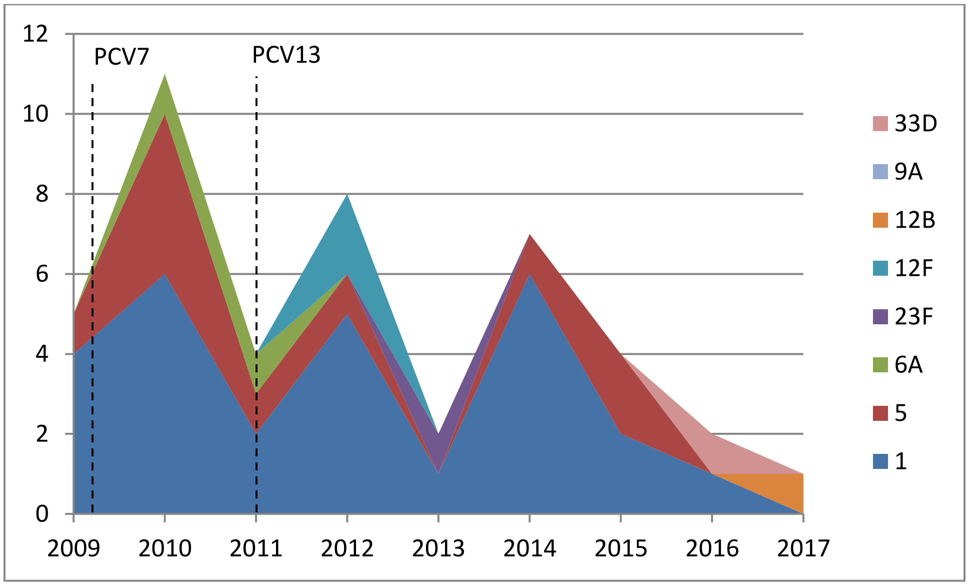

Figure S12. Annual counts of invasive pneumococcal disease due to PCV13 serotypes in children aged 2-11 months

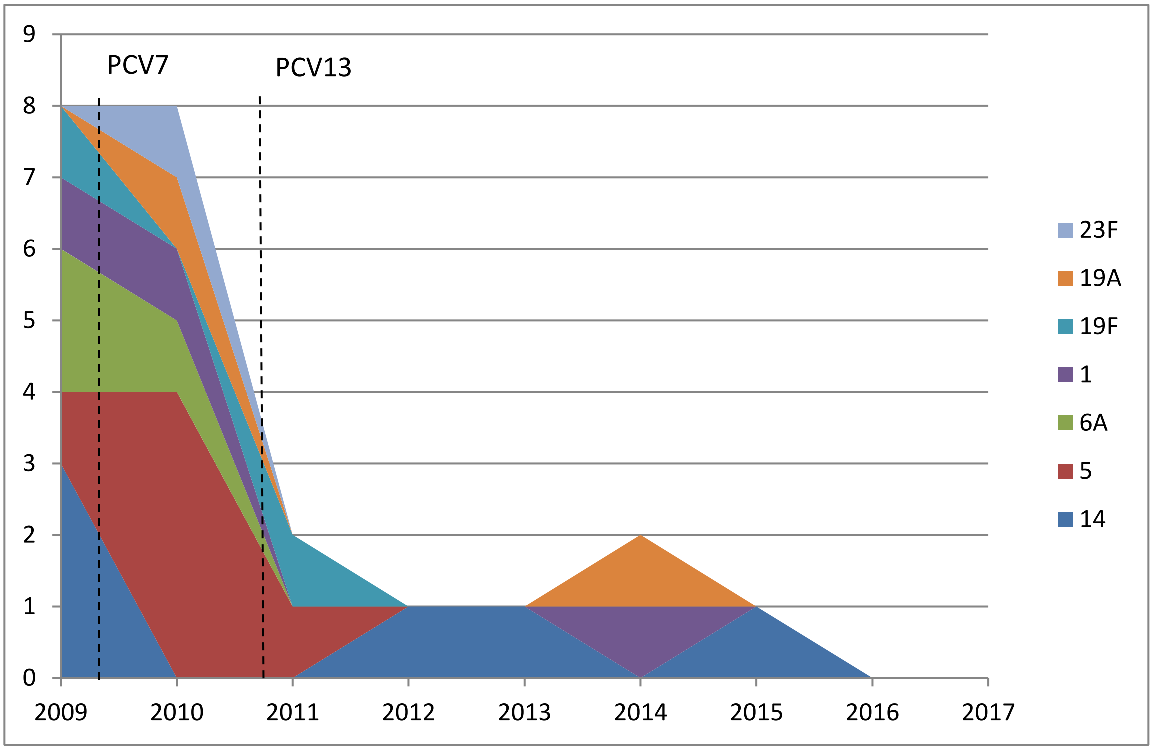

Note: Ten cases of non-vaccine type IPD were detected in the 2-11 month age group in the baseline period May 12, 2008 to May 11, 2010 and six cases were detected in 2016/17.

Figure S13. Adjusted annual incidence of radiological and clinical pneumonia in children aged 5-14 years between 2008 and 2017

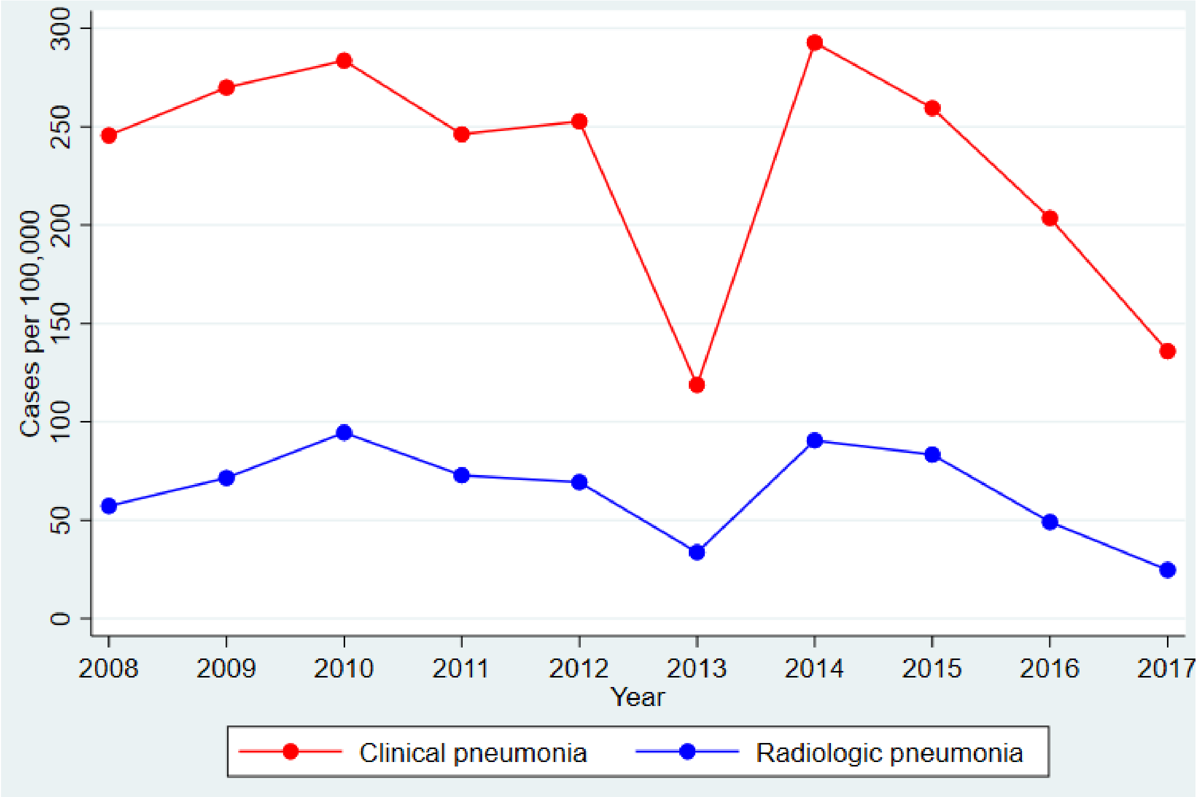

Figure S14. Proportion of patients aged 2 months to 14 years with suspected pneumonia, septicaemia, or meningitis in the wet season who had a positive malaria test, by year of the study

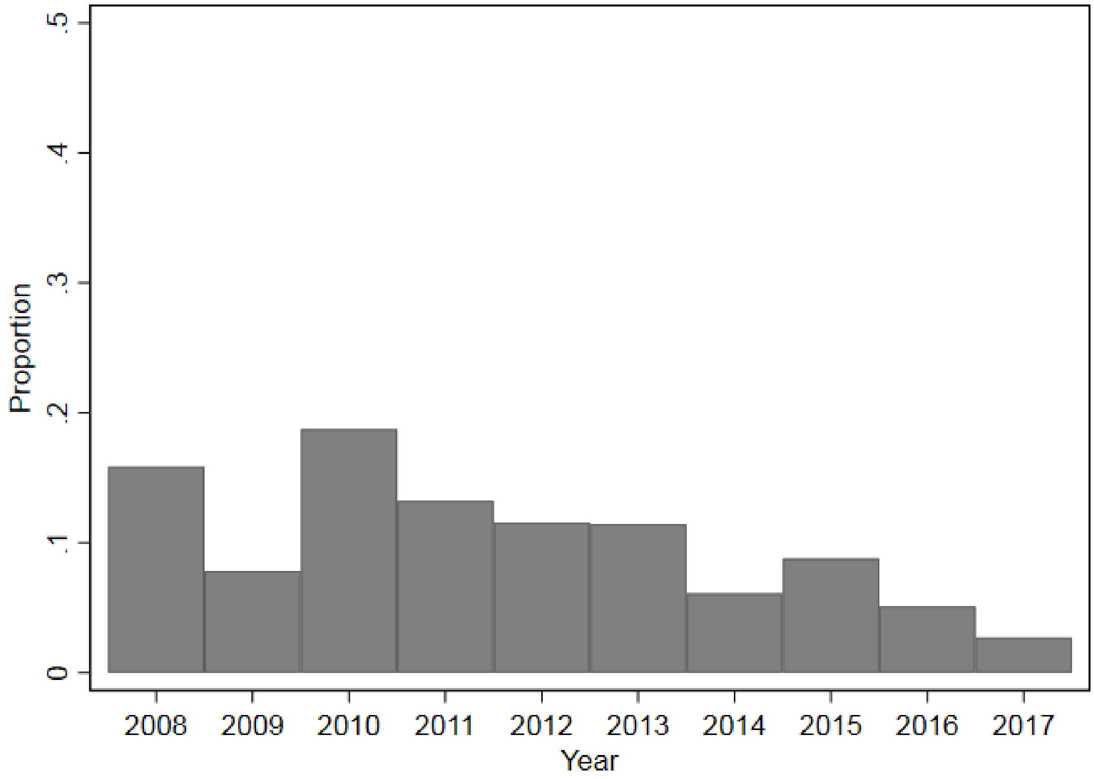

Figure S15. Proportion of children aged 2-59 months with suspected pneumonia, septicaemia, or meningitis who had weight-for-height z-score < -3, by year of the study

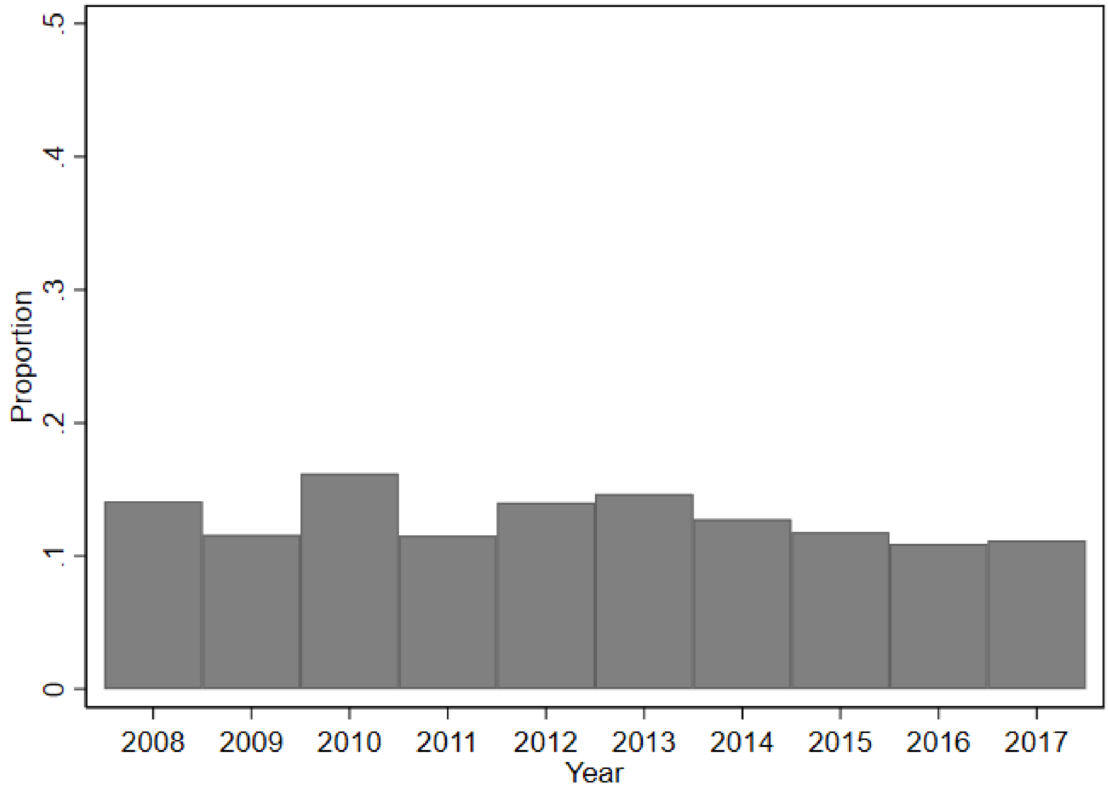

Figure S16. Proportion of children aged 2 months to 14 years with suspected pneumonia, septicaemia, or meningitis who had contaminated blood cultures, by year of the study

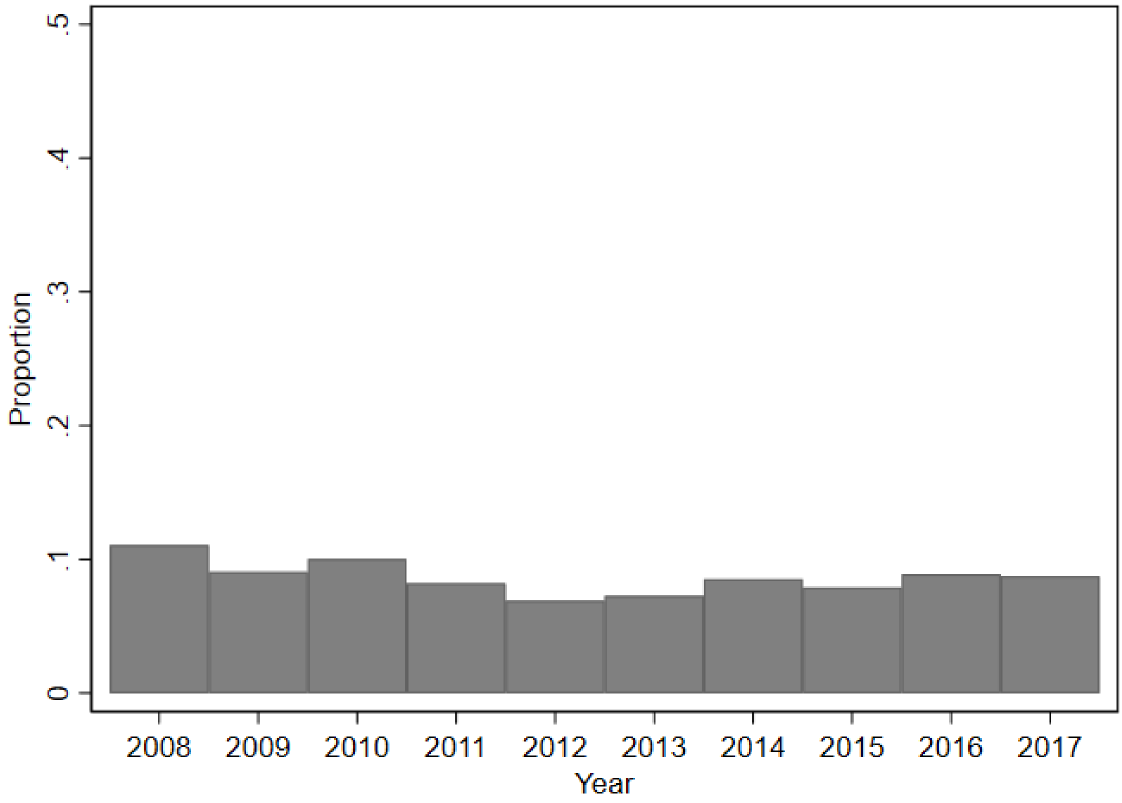

Figure S17. Adjusted annual incidence of bronchiolitis in young children between 2008 and 2017, by age group

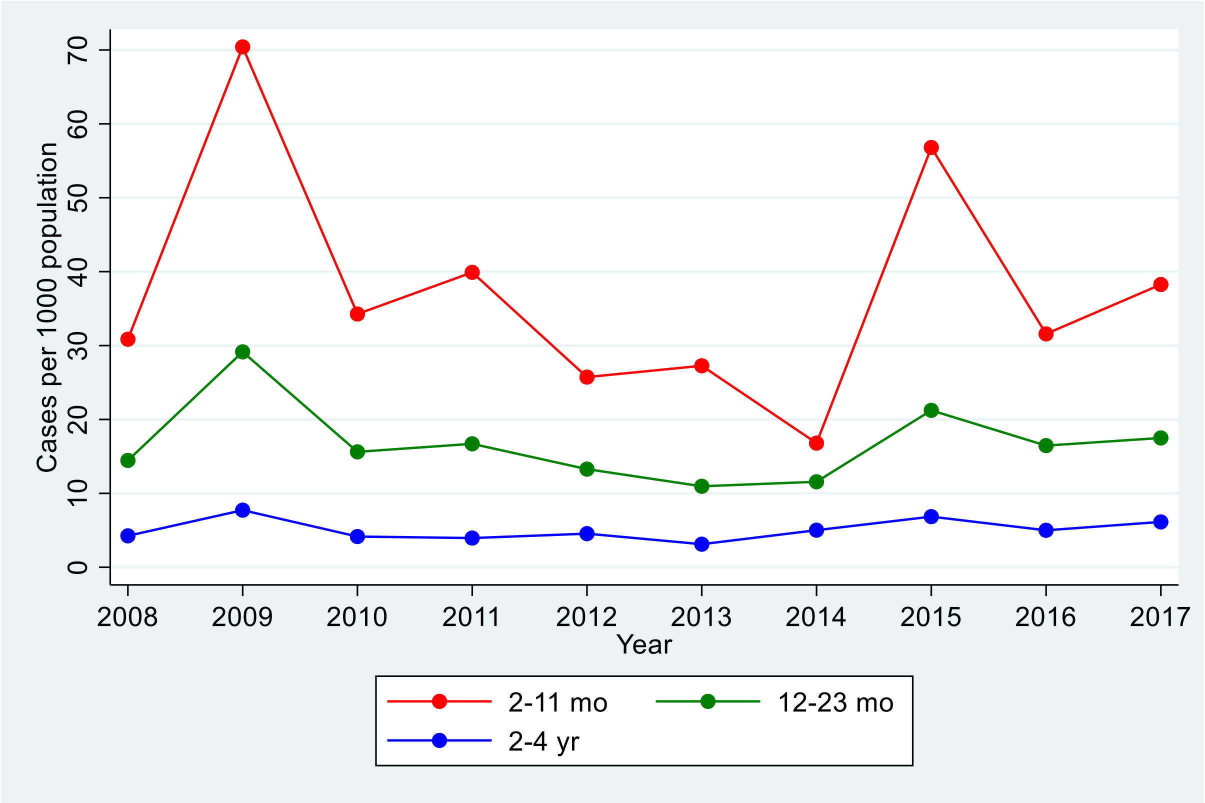

3. SUPPLEMENTARY TABLES

Table S1. Screening criteria for referral of out- and in-patients for clinician assessment (if one or more criteria are present for 14 days or less)

Patients aged 2 to 59 months

- History of cough or difficulty breathing, plus raised respiratory rate for age
- Lower chest wall indrawing, nasal flaring, or grunting
- Oxygen saturation less than 92%
- History of convulsion
- Impaired consciousness*
- Bulging fontanelle
- Stiff neck
- Axillary temperature at least 38°C, or less than 36°C, in a patient admitted or being admitted
- Prostration†
- Weight below -3 z score for age
- Local musculoskeletal swelling or tenderness
- Irrespective of age or residential location, any child with possible meningitis

Patients aged 5-14 years

- History of cough and difficulty breathing or pleuritic chest pain or supraclavicular/sternal recession or nasal flaring
- History of productive cough and fever
- History of convulsion
- Impaired consciousness *
- Altered mental state
- Photophobia
- Neck stiffness
- Axillary temperature of at least 38°C, or less than 36°C, in a patient admitted or being admitted
- History of rigors
- Local musculoskeletal swelling or tenderness
- **Irrespective of age or residential location, any patient with possible meningitis**

*Impaired consciousness is defined as V, P, or U on the AVPU score, where A is if the patient is alert, V if responsive to verbal stimulus, P if responsive to pain stimulus, and U if unresponsive. †Prostration is defined as an inability to drink or breast feed, or to remain in a seated position in a child otherwise able to do so.

Table S2. Clinical definitions for suspected pneumonia, septicaemia, and meningitis

	Age ≥2 months and <5 years	Age 5-14 years
**Suspected pneumonia**	Suspected pneumonia is defined if there is a history of cough or difficulty breathing of less than 14 days' duration, accompanied by one or more of:<br>1. Raised respiratory rate for age[*]<br>2. Lower chest wall indrawing, nasal flaring or grunting<br>3. Oxygen saturation less than 92%<br>4. Focal chest signs (dull percussion note, coarse crackles, bronchial breathing)	Suspected pneumonia will be defined according to clinical judgement and is to be considered in patients presenting with an illness of 14 days' duration or less, if two or more of the following are present::<br>1. Cough<br>2. Haemoptysis<br>3. Pleuritic chest pain<br>4. Breathlessness<br>5. Axillary temperature ≥38°C
**Suspected meningitis**	Suspected meningitis will be defined according to clinical judgement and is to be considered if any of the following are present:<br>1. Neck stiffness<br>2. Impaired consciousness[†]<br>3. Prostration[‡]<br>4. History of convulsion<br>5. Bulging fontanelle	Suspected meningitis will be defined according to clinical judgement and is to be considered if two or more of the following are present:<br>1. Axillary temperature ≥38°C<br>2. Meningism (neck stiffness and/or photophobia)<br>3. Altered mental state (Glasgow Coma Score <14)
**Suspected septicaemia**	Suspected septicaemia will be defined as one or more of:<br>1. Clinician diagnosis of focal sepsis (including but not limited to: septic arthritis, osteomyelitis, endocarditis, peritonitis, liver abscess, soft tissue abscess, cellulitis)<br>2. For a patient admitted, or being admitted, axillary temperature is <36°C or ≥38°C and no obvious cause of fever<br>3. For a patient admitted, or being admitted, the clinical impression is of severe malnutrition[§]	Suspected septicaemia will be defined as one or more of:<br>1. Clinician diagnosis of focal sepsis (including but not limited to: septic arthritis, osteomyelitis, endocarditis, peritonitis, liver abscess, soft tissue abscess, cellulitis)<br>2. For a patient admitted, or being admitted, axillary temperature is <36°C or ≥38°C and no obvious cause of fever<br>3. History of rigors

*Raised respiratory rate for age is defined as greater than 50 breaths per minute for children at least 2 months but less than 12 months, and as greater than 40 breaths per minute for children at least 12 months but less than 60 months.

†Impaired consciousness is defined as V, P, or U on the AVPU score, where A is if the patient is alert, V if responsive to verbal stimulus, P if responsive to pain stimulus, and U if unresponsive.

‡Prostration is defined as inability to drink or breast feed, or to remain in a seated position in a child otherwise able to do so.

§Severe malnutrition is defined according to the WHO definition.[10]

Table S3. Guideline for investigation of patients referred to clinicians and diagnosed with suspected pneumococcal disease according to clinical definitions

a. Patients with suspected pneumococcal disease are to have blood culture.
b. Patients with suspected meningitis are to have lumbar puncture.
c. Patients with suspected pneumonia are to have chest X-ray.
d. Chest X-ray should also be considered in patients with meningitis or septicaemia if the clinician's impression is of co-existing pneumonia or if it is judged that a chest X-ray will assist in management.
e. Lung aspirate should be considered for a patient if peripheral consolidation has been demonstrated, preferably by X-ray.
f. Other investigations including pleural tap and joint aspirate may be considered according to the clinical indication.
g. Patients with suspected pneumococcal disease are to have,
    i. a rapid diagnostic test for malaria (January – July; only if surveillance number ends in '0').
    ii. serum collection for antibiotic activity detection if surveillance number ends in '0' or '5' and the patient is enrolled in Basse.

Table S4. Crude and adjusted number of cases and incidence of IPD, excluding cases identified by lung aspiration alone, in the baseline period of May 12, 2008 to May 11, 2010 and in the 2016/17 period post-vaccine introduction, by age group and serotype

	5/2008–4/2010 adjusted (crude) cases	5/2008–4/2010 adjusted (crude) incidence per 100 000	2016/17 adjusted (crude) cases	2016/17 adjusted (crude) incidence per 100 000	Crude incidence rate ratio 2016/17 relative to 5/2008–4/2010 (95% CI)	Adjusted incidence rate ratio 2016/17 relative to 5/2008–4/2010 (95% CI)
**Age 2-23 months**						
All	56 (45)	253 (203)	19 (17)	67 (59)	0·29 (0·16–0·52)	0·26 (0·15–0·45)
PCV7	26 (21)	119 (95)	2 (2)	7 (7)	0·07 (0·02–0·33)	0·06 (0·01–0·26)
PCV13	44 (35)	197 (158)	6 (5)	20 (17)	0·11 (0·04–0·29)	0·10 (0·04–0·25)
PCV13 only	18 (15)	83 (68)	4 (3)	12 (10)	0·15 (0·04–0·54)	0·17 (0·06–0·52)
Non-vaccine type	12 (10)	56 (45)	14 (12)	48 (42)	0·91 (0·38–2·15)	0·85 (0·41–1·98)
**Age 2-4 years**						
All	34 (30)	107 (93)	5 (4)	11 (9)	0·10 (0·02–0·27)	0·10 (0·04–0·27)
PCV7	15 (13)	46 (40)	1 (1)	2 (2)	0·05 (0·01–0·42)	0·05 (0·007–0·37)
PCV13	30 (26)	92 (81)	3 (2)	6 (4)	0·05 (0·01–0·23)	0·06 (0·02–0·22)
PCV13 only	16 (14)	50 (43)	1 (1)	3 (2)	0·05 (0·01–0·39)	0·06 (0·01–0·35)
Non-vaccine type	5 (4)	15 (12)	3 (2)	6 (4)	0·36 (0·07–1·95)	0·38 (0·08–1·74)
**Age 5-14 years**						
All	6 (7)	7 (8)	4 (3)	3 (3)	0·33 (0·07–1·51)	0·51 (0·12–2·12)
PCV7	0 (0)	unsp.	0 (0)	unsp.	unsp.	unsp.
PCV13	5 (6)	6 (7)	1 (1)	1 (<1)	0·13 (0·01–1·39)	0·15 (0·01–1·72)
PCV13 only	5 (6)	6 (7)	1 (1)	<1 (<1)	0·13 (0·01–1·39)	0·15 (0·01–1·72)
Non-vaccine type	1 (1)	1 (1)	2 (2)	2 (2)	1·53 (0·10–22·9)	1·53 (0·10–22·9)

PCV7=serotypes covered by PCV7. PCV13=serotypes covered by PCV13. PCV13 only=serotypes covered by PCV13 but not PCV7. Non-vaccine type=serotypes not covered by PCV13. Exclusion of cases from adjusted counts used the age- and year-specific distributions of IPD cases identified by lung aspiration alone. Adjusted case counts are rounded to the nearest integer. Confidence intervals calculated taking into account over-dispersed Poisson distributions in the 2–23 months and 5–14 years age groups.

Table S5. Crude and adjusted number of cases and incidence of IPD, excluding cases treated as outpatients, in the baseline period of May 12, 2008 to May 11, 2010 and in the 2016/17 period post-vaccine introduction, by age group and serotype

	5/2008–4/2010 adjusted (crude) cases	5/2008–4/2010 adjusted (crude) incidence per 100 000	2016/17 adjusted (crude) cases	2016/17 adjusted (crude) incidence per 100 000	Crude incidence rate ratio 2016/17 relative to 5/2008–4/2010 (95% CI)	Adjusted incidence rate ratio 2016/17 relative to 5/2008–4/2010 (95% CI)
**Age 2-23 months**						
All	57 (46)	258 (208)	13 (12)	45 (42)	0·20 (0·10–0·39)	0·18 (0·09–0·33)
PCV7	27 (22)	124 (99)	1 (1)	4 (3)	0·03 (0·004–0·27)	0·03 (0·004–0·22)
PCV13	45 (36)	202 (162)	5 (4)	16 (14)	0·09 (0·03–0·28)	0·09 (0·03–0·22)
PCV13 only	18 (15)	83 (68)	4 (3)	12 (10)	0·15 (0·04–0·55)	0·17 (0·06–0·52)
Non-vaccine type	12 (10)	56 (45)	8 (8)	29 (28)	0·62 (0·24–1·60)	0·51 (0·20–1·29)
**Age 2-4 years**						
All	32 (28)	99 (87)	6 (5)	13 (11)	0·13 (0·05–0·33)	0·14 (0·06–0·32)
PCV7	12 (11)	39 (34)	0 (0)	unsp.	unsp.	unsp.
PCV13	28 (25)	88 (78)	3 (2)	6 (4)	0·06 (0·01–0·24)	0·06 (0·02–0·23)
PCV13 only	17 (15)	53 (47)	3 (2)	6 (4)	0·10 (0·02–0·42)	0·10 (0·03–0·39)
Non-vaccine type	4 (3)	11 (9)	4 (3)	8 (7)	0·71 (0·14–3·53)	0·72 (0·17–3·11)
**Age 5-14 years**						
All	7 (8)	8 (9)	4 (3)	3 (3)	0·29 (0·06–1·28)	0·44 (0·11–1·75)
PCV7	0 (0)	unsp.	0 (0)	unsp.	unsp.	unsp.
PCV13	7 (8)	8 (9)	1 (1)	1 (<1)	0·10 (0·01–1·00)	0·11 (0·01–1·16)
PCV13 only	7 (8)	8 (9)	1 (1)	1 (<1)	0·10 (0·01–1·00)	0·11 (0·01–1·16)
Non-vaccine type	0 (0)	unsp.	2 (2)	2 (2)	unsp.	unsp.

PCV7=serotypes covered by PCV7. PCV13=serotypes covered by PCV13. PCV13 only=serotypes covered by PCV13 but not PCV7. Non-vaccine type=serotypes not covered by PCV13. Exclusion of cases from adjusted counts used the age- and year-specific distributions of outpatient cases of IPD. Adjusted case counts are rounded to the nearest integer. Confidence intervals calculated taking into account over-dispersed Poisson distributions in the 2–23 months and 5–14 years age groups.

Table S6. Crude and adjusted number of cases and incidence of IPD, excluding cases of serotype 1 or 5, in the baseline period of May 12, 2008 to May 11, 2010 and in the 2016/17 period post-vaccine introduction, by age group and serotype

	5/2008–4/2010 adjusted (crude) cases	5/2008–4/2010 adjusted (crude) incidence per 100 000	2016/17 adjusted (crude) cases	2016/17 adjusted (crude) incidence per 100 000	Crude incidence rate ratio 2016/17 relative to 5/2008–4/2010 (95% CI)	Adjusted incidence rate ratio 2016/17 relative to 5/2008–4/2010 (95% CI)
**Age 2-23 months**						
All	46 (37)	209 (167)	18 (16)	64 (56)	0·33 (0·18–0·61)	0·31 (0·17–0·53)
PCV7	29 (23)	129 (104)	2 (2)	7 (7)	0·07 (0·02–0·30)	0·06 (0·01–0·23)
PCV13	31 (25)	141 (113)	4 (3)	12 (10)	0·09 (0·03–0·32)	0·09 (0·03–0·29)
PCV13 only	3 (2)	12 (9)	1 (1)	5 (3)	0·38 (0·03–4·54)	0·43 (0·–4·)
Non-vaccine type	15 (12)	67 (54)	15 (13)	51 (45)	0·83 (0·37–1·86)	0·76 (0·37–1·61)
**Age 2-4 years**						
All	22 (19)	68 (59)	7 (6)	16 (13)	0·23 (0·09–0·56)	0·23 (0·10–0·54)
PCV7	15 (13)	46 (40)	1 (1)	2 (2)	0·05 (0·007–0·42)	0·05 (0·007–0·37)
PCV13	17 (15)	53 (47)	4 (3)	8 (7)	0·14 (0·04–0·49)	0·15 (0·05–0·47)
PCV13 only	2 (2)	7 (6)	3 (2)	6 (4)	0·71 (0·10–5·06)	0·76 (0·13–4·49)
Non-vaccine type	5 (4)	15 (12)	4 (3)	8 (7)	0·54 (0·12–2·39)	0·54 (0·14–2·11)
**Age 5-14 years**						
All	1 (1)	1 (1)	2 (2)	2 (2)	1·53 (0·10–22·9)	1·53 (0·10–22·9)
PCV7	0 (0)	unsp.	0 (0)	unsp.	unsp.	unsp.
PCV13	0 (0)	unsp.	0 (0)	unsp.	unsp.	unsp.
PCV13 only	0 (0)	unsp.	0 (0)	unsp.	unsp.	unsp.
Non-vaccine type	1 (1)	1 (1)	2 (2)	2 (2)	1·53 (0·10 22·9)	1·53 (0·10–22.9)

PCV7=serotypes covered by PCV7. PCV13=serotypes covered by PCV13. PCV13 only=serotypes covered by PCV13 but not PCV7. Non-vaccine type=serotypes not covered by PCV13. Exclusion of cases from adjusted counts used the age-, year-, and serotype category-specific distributions of IPD case due to serotypes 1 or 5. Adjusted case counts are rounded to the nearest integer. Confidence intervals calculated taking into account over-dispersed Poisson distributions in the 2–23 months and 5–14 years age groups.

Table S7. Crude and adjusted number of cases and incidence of non-pneumococcal bacteremia in the baseline period of May 12, 2008 to May 11, 2010 and the 2016/17 period post-vaccine introduction, by age group

Age group	5/2008–4/2010 adjusted (crude) cases	5/2008–4/2010 adjusted (crude) incidence per 100 000	2016/17 adjusted (crude) cases	2016/17 adjusted (crude) incidence per 100 000	Crude incidence rate ratio 2016/17 relative to 5/2008–4/2010 (95% CI)	Adjusted incidence rate ratio 2016/17 relative to 5/2008–4/2010 (95% CI)
2–23 months	57 (45)	256 (203)	62 (51)	216 (177)	0·87 (0·58–1·32)	0·84 (0·58–1·21)
2–4 years	12 (10)	38 (31)	20 (16)	45 (35)	1·14 (0·52–2·52)	1·19 (0·59–2·42)
2–59 months	69 (55)	127 (101)	83 (67)	112 (91)	0·90 (0·63–1·28)	0·88 (0·64–1·21)
5–14 years	4 (5)	5 (6)	7 (5)	6 (4)	0·77 (0·19–3·10)	1·16 (0·34–3·91)

Adjusted case counts take account of changes over time in the sensitivity of case ascertainment, and are rounded to the nearest integer. Confidence intervals calculated taking account of over-dispersed Poisson distributions in the 2–23 months and 5–14 years age groups. Numbers of individual bacteraemia cases throughout the observation period: *E. coli* (n=45), *Acinetobacter* sp. (n=8), *H. influenzae* type b (n=24), *H. influenzae* non-type b (n=17), *Klebsiella* sp. (n=11), Non-typhi salmonella (n=48), *Staphylococcus aureus* (n=78), *Pseudomonas* sp. (n=21), Other gram positive (n=38), Other gram negative (n=86).

Table S8. Characteristics of children with invasive pneumococcal disease and vaccine failure

Count	Date onset of illness	Age (mo)	Suspected diagnosis	Weight-for-Height z-score <-3	Child HIV infection	Mother HIV infection	Serotype	Clinical sample	Vaccine	No. doses prior to illness	No. months since last dose
1	27 May 2010	14	Pneumonia	Yes	ND	ND	19F	LA	PCV7	3	5
2	15 June 2010	12	Pneumonia	No	ND	ND	14	LA	PCV7	3	7
3	11 Nov 2010	15	Pneumonia	No	ND	ND	6A	LA & BC	PCV7	3	8
4	7 May 2012	16	Pneumonia	Yes	ND	ND	19F	BC	PCV7	3	11
5	2 Dec 2012	25	Meningitis	No	ND	ND	23F	BC	PCV7	3	7
6	8 Jan 2013	27	Meningitis	No	ND	ND	23F	CSF & BC	PCV7	2	18
7	2 Feb 2013	32	Pneumonia	No	No	No	23F	BC	PCV7	3	26
8	11 Feb 2013	16	Pneumonia	No	Yes	Yes	14	BC	PCV13	3	10
9	25 Feb 2013	20	Sepsis	No	ND	ND	23F	BC	PCV13	2	12
10	14 Mar 2013	30	Pneumonia	No	No	ND	1/6A/B	BC	PCV7	3	23
11	4 Aug 2013	18	Pneumonia	No	No	No	1	LA	PCV13	3	11
12	5 Aug 2013	11	Pneumonia	No	No	ND	14	BC	PCV13	3	8
13	26 Aug 2013	19	Pneumonia	No	No	ND	19A	BC	PCV13	3	17
14	6 Mar 2014	17	Meningitis	No	No	No	14	BC	PCV13	3	8
15	17 Mar 2014	19	Pneumonia	Yes	No	ND	14	BC	PCV13	2	14
16	5 Sep 2014	26	Pneumonia	No	No	ND	1	BC	PCV13	3	20
17	2 Nov 2014	24	Sepsis	Yes	No	No	19A	BC	PCV13	3	17
18	23 Mar 2015	27	Sepsis	No	No	No	5	BC	PCV13	3	18
19	24 Jul 2015	9	Pneumonia	No	No	No	14	BC	PCV13	3	4
20	15 Oct 2015	16	Sepsis	Yes	ND	ND	19A	BC	PCV13	3	11
21	13 Nov 2015	20	Sepsis	No	ND	ND	14	LA	PCV13	3	15
22	24 Feb 2016	22	Sepsis	No	No	No	23F	BC	PCV13	3	18
23	25 Feb 2016	46	Sepsis	No	ND	ND	3	LA	PCV13	3	39
24	7 Mar 2016	42	Pneumonia	No	ND	ND	14	BC	PCV13	3	36
25	19 Mar 2016	19	Pneumonia	Yes	ND	ND	9V	BC	PCV13	3	12
26	25 Mar 2016	23	Sepsis	No	No	No	5	BC	PCV13	3	16
27	2 Jun 2016	22	Pneumonia	No	ND	ND	1	BC	PCV13	3	15

Table S9. Crude and adjusted number of cases and incidence of pneumococcal pneumonia endpoints, excluding cases identified by lung aspiration alone, in the baseline period of 12 May 2008 to 11 May 2010 and in the 2016/17 period post-vaccine introduction, by age group

	5/2008–4/2010 adjusted (crude) cases	5/2008–4/2010 adjusted (crude) incidence per 1000 person-years	2016/17 adjusted (crude) cases	2016/17 adjusted (crude) incidence per 1000 person-years	Crude incidence rate ratio 2016/17 relative to 5/2008–4/2010 (95% CI)	Adjusted incidence rate ratio 2016/17 relative to 5/2008–4/2010 (95% CI)
**Age 2-11 months**						
Radiological pneumonia						
Pneumococcal	19 (11)	1·8 (1·1)	2 (2)	0·2 (0·2)	0·15 (0·03–0·66)	0·09 (0·02–0·38)
PCV13 vaccine-type	10 (6)	1·0 (0·6)	0 (0)	unsp.	unsp.	unsp.
Non-vaccine-type	8 (5)	0·8 (0·5)	2 (2)	0·2 (0·2)	0·32 (0·06–1·65)	0·21 (0·05–0·95)
Clinical pneumonia						
Pneumococcal	38 (23)	3·7 (2·2)	5 (5)	0·4 (0·4)	0·17 (0·07–0·46)	0·11 (0·04–0·28)
PCV13 vaccine-type	23 (14)	2·2 (1·3)	0 (0)	unsp.	unsp.	unsp.
Non-vaccine-type	14 (9)	1·3 (0·9)	5 (5)	0·4 (0·4)	0·44 (0·15–1·32)	0·30 (0·11–0·82)
**Age 12-23 months**						
Radiological pneumonia						
Pneumococcal	32 (21)	2·6 (1·7)	8 (7)	0·5 (0·4)	0·26 (0·11–0·61)	0·20 (0·10–0·43)
PCV13 vaccine-type	30 (20)	2·4 (1·6)	5 (4)	0·3 (0·3)	0·16 (0·05–0·46)	0·12 (0·05–0·33)
Non-vaccine-type	2 (1)	0·2 (0·08)	4 (3)	0·3 (0·2)	2·35 (0·24–22·6)	1·18 (0·23–5·94)
Clinical pneumonia						
Pneumococcal	36 (23)	2·9 (1·9)	15 (12)	1·0 (0·8)	0·41 (0·20–0·82)	0·32 (0·18–0·59)
PCV13 vaccine-type	32 (22)	2·6 (1·8)	6 (5)	0·4 (0·3)	0·18 (0·07–0·47)	0·14 (0·06–0·34)
Non-vaccine-type	4 (1)	0·3 (0·08)	9 (7)	0·6 (0·4)	5·49 (0·67–44·6)	1·94 (0·58–6·56)
**Age 2-4 years**						
Radiological pneumonia						
Pneumococcal	27 (21)	0·8 (0·7)	4 (3)	0·09 (0·07)	0·10 (0·03–0·39)	0·11 (0·03–0·39)
PCV13 vaccine-type	24 (18)	0·7 (0·6)	1 (1)	0·02 (0·02)	0·04 (0·004–0·37)	0·03 (0·004–0·37)
Non-vaccine-type	4 (3)	0·1 (0·09)	3 (2)	0·07 (0·04)	0·46 (0·07–3·43)	0·53 (0·07–3·43)
Clinical pneumonia						
Pneumococcal	41 (30)	1·3 (0·9)	5 (4)	0·1 (0·09)	0·10 (0·03–0·30)	0·09 (0·03–0·30)
PCV13 vaccine-type	36 (26)	1·1 (0·8)	3 (2)	0·07 (0·04)	0·05 (0·01–0·27)	0·06 (0·01–0·27)
Non-vaccine-type	5 (4)	0·2 (0·1)	3 (2)	0·07 (0·04)	0·36 (0·05–2·32)	0·43 (0·05–2·32)
**Age 5-14 years**						
Radiological pneumonia						
Pneumococcal	6 (6)	0·07 (0·07)	4 (3)	0·04 (0·03)	0·38 (0·10–1·53)	0·57 (0·16–2·05)
PCV13 vaccine-type	6 (6)	0·07 (0·07)	1 (1)	0·01 (0·01)	0·13 (0·02–1·06)	0·17 (0·02–1·21)
Non-vaccine-type	0 (0)	unsp.	3 (2)	0·03 (0·02)	unsp.	unsp.
Clinical pneumonia						
Pneumococcal	6 (7)	0·07 (0·08)	4 (3)	0·04 (0·03)	0·33 (0·08–1·27)	0·50 (0·14–1·74)
PCV13 vaccine-type	6 (6)	0·07 (0·07).	1 (1)	0·01 (0·01)	0·13 (0·02–1·06)	0·17 (0·02–1·21)
Non-vaccine-type	1 (1)	0·01 (0·01)	3 (2)	0·03 (0·02)	1·53 (0·14–16·9)	2·91 (0·23–36·4)

PCV13=serotypes covered by PCV13. Non-vaccine type=serotypes not covered by PCV13. Exclusion of cases from adjusted counts used the age- and year-specific distributions of cases identified by lung aspiration alone. Adjusted case counts are rounded to the nearest integer. Confidence intervals calculated taking into account over-dispersed Poisson distributions in the 2–4 years age group.

Table S10. Crude and adjusted number of cases and incidence of pneumococcal pneumonia endpoints, excluding cases of serotype 1 or 5, in the baseline period of 12 May 2008 to 11 May 2010 and in the 2016/17 period post-vaccine introduction, by age group and serotype

	5/2008–4/2010 adjusted (crude) cases	5/2008–4/2010 adjusted (crude) incidence per 1000 person-years	2016/17 adjusted (crude) cases	2016/17 adjusted (crude) incidence per 1000 person-years	Crude incidence rate ratio 2016/17 relative to 5/2008–4/2010 (95% CI)	Adjusted incidence rate ratio 2016/17 relative to 5/2008–4/2010 (95% CI)
**Age 2-11 months**						
Radiological pneumonia						
Pneumococcal	12 (10)	1·2 (1·0)	3 (3)	0·2 (0·2)	0·24 (0·07–0·87)	0·21 (0·06–0·73)
PCV13 vaccine-type	5 (4)	0·5 (0·4)	0 (0)	unsp.	unsp.	unsp.
Non-vaccine-type	7 (6)	0·7 (0·6)	3 (3)	0·2 (0·2)	0·40 (0·10–1·60)	0·36 (0·09–1·35)
Clinical pneumonia						
Pneumococcal	25 (21)	2·4 (2·0)	6 (6)	0·5 (0·5)	0·23 (0·09–0·57)	0·20 (0·08–0·48)
PCV13 vaccine-type	13 (11)	1·2 (1·1)	0 (0)	unsp.	unsp.	unsp.
Non-vaccine-type	12 (10)	1·2 (1·0)	6 (6)	0·5 (0·5)	0·48 (0·17–1·32)	0·42 (0·16–1·11)
**Age 12-23 months**						
Radiological pneumonia						
Pneumococcal	19 (15)	1·5 (1·2)	6 (5)	0·4 (0·3)	0·26 (0·09–0·72)	0·26 (0·11–0·64)
PCV13 vaccine-type	16 (13)	1·3 (1·1)	3 (2)	0·2 (0·1)	0·12 (0·03–0·53)	0·13 (0·03–0·46)
Non-vaccine-type	3 (2)	0·2 (0·2)	4 (3)	0·3 (0·2)	1·18 (0·19–7·03)	1·13 (0·23–5·59)
Clinical pneumonia						
Pneumococcal	19 (15)	1·5 (1·2)	13 (10)	0·8 (0·6)	0·52 (0·23–1·16)	0·53 (0·26–1·07)
PCV13 vaccine-type	16 (13)	1·3 (1·1)	4 (3)	0·3 (0·2)	0·18 (0·05–0·63)	0·18 (0·06–0·54)
Non-vaccine-type	3 (2)	0·2 (0·2)	9 (7)	0·6 (0·4)	2·74 (0·57–13·2)	2·78 (0·69–11·1)
**Age 2-4 years**						
Radiological pneumonia						
Pneumococcal	14 (12)	0·4 (0·4)	6 (5)	0·1 (0·1)	0·30 (0·09–0·94)	0·31 (0·11–0·88)
PCV13 vaccine-type	10 (9)	0·3 (0·3)	2 (2)	0·04 (0·04)	0·16 (0·03–0·86)	0·14 (0·03–0·76)
Non-vaccine-type	4 (3)	0·1 (0·09)	4 (3)	0·09 (0·07)	0·71 (0·12–4·17)	0·71 (0·15–3·29)
Clinical pneumonia						
Pneumococcal	21 (18)	0·7 (0·6)	7 (6)	0·2 (0·1)	0·24 (0·09–0·66)	0·23 (0·09–0·58)
PCV13 vaccine-type	16 (14)	0·5 (0·4)	4 (3)	0·09 (0·07)	0·15 (0·04–0·61)	0·18 (0·05–0·60)
Non-vaccine type	5 (4)	0·2 (0·1)	4 (3)	0·09 (0·07)	0·53 (0·10–2·79)	0·57 (0·13–2·44)
**Age 5-14 years**						
Radiological pneumonia						
Pneumococcal	0 (0)	unsp.	3 (2)	0·03 (0·02)	unsp.	unsp.
PCV13 vaccine-type	0 (0)	unsp.	0 (0)	unsp.	unsp.	unsp.
Non-vaccine-type	0 (0)	unsp.	3 (2)	0·03 (0·02)	unsp.	unsp.
Clinical pneumonia						
Pneumococcal	1 (1)	0·01 (0·01)	3 (2)	0·03 (0·02)	1·53 (0·14–16·9)	2·9 (0·23–36·4)
PCV13 vaccine-type	0 (0)	unsp.	0 (0)	unsp.	unsp.	unsp.
Non-vaccine-type	1 (1)	0·01 (0·01)	3 (2)	0·03 (0·02)	1·53 (0·14–16·9)	2·9 (0·23–36·4)

PCV13=serotypes covered by PCV13. Non-vaccine type=serotypes not covered by PCV13. Exclusion of cases from adjusted counts used the age-, year-, and serotype category-specific distributions of case due to serotypes 1 or 5. Adjusted case counts are rounded to the nearest integer. Confidence intervals calculated taking into account overdispersed Poisson distributions in the 2–4 years age group.

Table S11. Crude and adjusted number of cases and incidence of clinical pneumonia caused by non-pneumococcal bacteria in the baseline period of 12 May 2008 to 11 May 2010 and the 2016/17 period post-vaccine introduction, by age group

Age group	5/2008–4/2010 adjusted (crude) cases	5/2008–4/2010 adjusted (crude) incidence per 1000 person-years	2016/17 adjusted (crude) cases	2016/17 adjusted (crude) incidence per 1000 person-years	Crude incidence rate ratio 2016/17 relative to 5/2008–4/2010 (95% CI)	Adjusted incidence rate ratio 2016/17 relative to 5/2008–4/2010 (95% CI)
2–11 months	26 (22)	2·5 (2·1)	41 (32)	3·2 (2·5)	1·16 (0·68–2·00)	1·23 (0·75–2·00)
12-23 months	28 (22)	2·3 (1·8)	17 (15)	1·1 (1·0)	0·53 (0·28–1·03)	0·48 (0·26–0·87)
2–4 years	12 (10)	0·4 (0·3)	20 (15)	0·4 (0·3)	1·07 (0·44–2·59)	1·19 (0·54–2·62)
2–59 months	66 (54)	1·2 (1·0)	78 (62)	1·1 (0·8)	0·85 (0·59–1·23)	0·87 (0·63–1·21)
5–14 years	2 (2)	0·02 (0·02)	5 (4)	0·04 (0·04)	1·53 (0·28–8·37)	1·98 (0·36–10·8)

Adjusted case counts take account of changes over time in the rate of patients meeting criteria for referral, and are rounded to the nearest integer.

Table S12. Crude and adjusted number of cases and incidence of bronchiolitis in the baseline period of 12 May 2008 to 11 May 2010 and the 2016/17 period post-vaccine introduction, by age group

Age group	5/2008–4/2010 adjusted (crude) cases	5/2008–4/2010 adjusted (crude) incidence per 1000 person-years	2016/17 adjusted (crude) cases	2016/17 adjusted (crude) incidence per 1000 person-years	Crude incidence rate ratio 2016/17 relative to 5/2008–4/2010 (95% CI)	Adjusted incidence rate ratio 2016/17 relative to 5/2008–4/2010 (95% CI)
2–11 months	528 (441)	50·8 (42·4)	455 (359)	35·0 (27·6)	0·65 (0·57–0·75)	0·69 (0·61–0·78)
12–23 months	265 (208)	21·4 (16·8)	268 (210)	17·0 (13·3)	0·79 (0·65–0·96)	0·79 (0·67–0·94)
2–4 years	197 (169)	6·1 (5·2)	252 (194)	5·6 (4·3)	0·82 (0·67–1·01)	0·91 (0·74–1·12)

Adjusted case counts take account of changes over time in the rate of patients meeting criteria for referral, and are rounded to the nearest integer. Bronchiolitis was defined as clinical pneumonia with auscultatory wheeze without radiologic pneumonia, dullness to percussion or bronchial breathing.

4. REFERENCES

1. National AIDS Control Programme. Republic of The Gambia, HIV Sentinel Surveillance Report 2011. Banjul: Ministry of Health & Social Welfare, 2012

2. World Health Organization Pneumonia Vaccine Trial Investigators' Group. Standardization of interpretation of chest radiographs for the diagnosis of pneumonia in children. World Health Organization, Department of Vaccines and Biologicals, 2001 (Accessed October 3, 2004 www.who.int/vaccines-documents/).

3. Adegbola RA, Falade AG, Sam BE et al. The etiology of pneumonia in malnourished and well-nourished Gambian children. Pediatr Infect Dis J 1994;13(11):975-982.

4. Mackenzie GA, Hill PC, Jeffries DJ et al. Effect of the introduction of pneumococcal conjugate vaccination on invasive pneumococcal disease in The Gambia: a population-based surveillance study. Lancet Infect Dis 2016;16(6):703-711.

5. Mackenzie GA, Hill PC, Sahito SM et al. Impact of the introduction of pneumococcal conjugate vaccination on pneumonia in The Gambia: population-based surveillance and case-control studies. Lancet Infect Dis 2017;17(9):965-973.

6. Miller E, Andrews NJ, Waight PA, Slack MP, George RC. Herd immunity and serotype replacement 4 years after seven-valent pneumococcal conjugate vaccination in England and Wales: an observational cohort study. Lancet Infect Dis 2011;11(10):760-768.

7. Waight PA, Andrews NJ, Ladhani SN, Sheppard CL, Slack MP, Miller E. Effect of the 13-valent pneumococcal conjugate vaccine on invasive pneumococcal disease in England and Wales 4 years after its introduction: an observational cohort study. Lancet Infect Dis 2015;15(5):535-543.

8. Greenland S, Rothman KJ. Introduction to stratified analysis. In: Rothman KJ, Greenland S, Lash TL, eds. Modern Epidemiology. 3rd ed. Philadelphia, USA: Lippincott Williams & Wilkins; 2008:258-282.
